# Kinetics of Physiological Responses as a Measure of Intensity and Hydration Status During Experimental Physical Stress in Human Volunteers

**DOI:** 10.3389/fphys.2020.01006

**Published:** 2020-09-04

**Authors:** Shirley W. Kartaram, Klaske van Norren, Eric Schoen, Marc Teunis, Marco Mensink, Martie Verschuren, Laura M’Rabet, Isolde Besseling-van der Vaart, Karin Mohrmann, Harriet Wittink, Johan Garssen, Renger Witkamp, Raymond Pieters

**Affiliations:** ^1^Research Group Innovative Testing in Life Sciences and Chemistry, University of Applied Sciences Utrecht, Utrecht, Netherlands; ^2^Department of Nutritional Biology, Division of Human Nutrition and Health, Wageningen University & Research, Wageningen, Netherlands; ^3^Netherlands Organization for Applied Scientific Research, Zeist, Netherlands; ^4^Research Group Analysis Techniques in Life Sciences, Avans University of Applied Sciences, Breda, Netherlands; ^5^Winclove Probiotics B.V., Amsterdam, Netherlands; ^6^Star-shl, Etten-Leur, Netherlands; ^7^Research Group Lifestyle and Health, University of Applied Sciences Utrecht, Utrecht, Netherlands; ^8^Department of Pharmaceutical Sciences, Utrecht University, Utrecht, Netherlands; ^9^Nutricia Research, Utrecht, Netherlands; ^10^Institute for Risk Assessment Sciences (IRAS), Utrecht University, Utrecht, Netherlands

**Keywords:** kinetics, biomarkers, exercise-intensity, resilience, dehydration, physiological responses

## Abstract

**Introduction:**

Strenuous physical stress induces a range of physiological responses, the extent depending, among others, on the nature and severity of the exercise, a person’s training level and overall physical resilience. This principle can also be used in an experimental set-up by measuring time-dependent changes in biomarkers for physiological processes. In a previous report, we described the effects of workload delivered on a bicycle ergometer on intestinal functionality. As a follow-up, we here describe an analysis of the kinetics of various other biomarkers.

**Aim:**

To analyse the time-dependent changes of 34 markers for different metabolic and immunological processes, comparing four different exercise protocols and a rest protocol.

**Methods:**

After determining individual maximum workloads, 15 healthy male participants (20–35 years) started with a rest protocol and subsequently performed (in a cross-over design with 1-week wash-out) four exercise protocols of 1-h duration at different intensities: 70% W_*max*_ in a hydrated and a mildly dehydrated state, 50% W_*max*_ and intermittent 85/55% W_*max*_ in blocks of 2 min. Perceived exertion was monitored using the Borg’ Rating of Perceived Exertion scale. Blood samples were collected both before and during exercise, and at various timepoints up to 24 h afterward. Data was analyzed using a multilevel mixed linear model with multiple test correction.

**Results:**

Kinetic changes of various biomarkers were exercise-intensity-dependent. Biomarkers included parameters indicative of metabolic activity (e.g., creatinine, bicarbonate), immunological and hematological functionality (e.g., leukocytes, hemoglobin) and intestinal physiology (citrulline, intestinal fatty acid-binding protein, and zonulin). In general, responses to high intensity exercise of 70% W_*max*_ and intermittent exercise i.e., 55/85% W_*max*_ were more pronounced compared to exercise at 50% W_*max*_.

**Conclusion:**

High (70 and 55/85% W_*max*_) and moderate (50% W_*max*_) intensity exercise in a bicycle ergometer test produce different time-dependent changes in a broad range of parameters indicative of metabolic activity, immunological and hematological functionality and intestinal physiology. These parameters may be considered biomarkers of homeostatic resilience. Mild dehydration intensifies these time-related changes. Moderate intensity exercise of 50% W_*max*_ shows sufficient physiological and immunological responses and can be employed to test the health condition of less fit individuals.

## Introduction

Current definitions of health are increasingly based on the concept that health should be considered a dynamic condition which is largely determined by the resilience of individuals to cope with stressful conditions that might disturb homeostasis (Feder et al., 2009; Huber et al., 2011, 2012). For example, a recent definition describes health as “the ability to adapt and to self-manage, in the face of social, physical and emotional challenges” (Huber et al., 2011). This concept also implies that information on the health status of an individual can be inferred from the kinetics of a physiological or psychological response to a stressor. Obviously, the scope of that information does not cover all elements of health and depends on the nature of the test that is applied, the data collected and their interpretation.

The principle to evaluate homeostatic resilience by measuring the response to a particular stressor is relevant, for instance, to monitor the outcome of interventions aimed at improving someone’s health status.

Suitable human test models to study resilience use different stressors, such as vaccination (to study immune responsiveness) (Petry et al., 1991; Michel et al., 1997), a test meal high in fat, carbohydrates or other nutrient combinations to study metabolic resilience (Wopereis et al., 2013; Kardinaal et al., 2015) or physical exercise (Lamprecht et al., 2012; JanssenDuijghuijsen et al., 2016, 2017a).

Many studies have examined physiological responses to physical exercise as stressor, in particular with regard to sport performance and training. Raynaud et al. (1997) proposed a cycling model where volunteers performed at 90% of the calculated W_*max*_, based on the age of the volunteer. The authors measured different parameters including bicarbonate, creatine kinase, potassium, chloride and sodium (Raynaud et al., 1997). Shek et al. (1995) and Kakanis et al. (2010) studied immune changes at multiple timepoints, both during and after strenuous endurance exercise, in well-trained athletes. These investigators demonstrated that exercise caused a rapid but temporal deviation of baseline leukocyte counts, including those of neutrophils and lymphocytes, and observed a return to baseline level within 24 h post exercise. In a previous study carried out by some of the co-authors of the present paper (Carol et al., 2011), similar effects were seen on neutrophil counts, as well as typical changes in cytokine levels after an exercise challenge consisting of 90 min of cycling at 50% W_*max*_ by young men who were in a glycogen-depleted state. A series of follow-up studies (JanssenDuijghuijsen et al., 2016, 2017b) focused on intestinal permeability in response to strenuous exercise. The outcome of these latter studies underlined that changes in gut barrier function play important roles in the physiological responses to exercise. But although studies demonstrate kinetics of immuno-physiological responses to exercise, none of these studies has specifically addressed the kinetics of physiological responses as function of the extent of physical stress and as a possible measure of health in non-trained individuals. Hence, insight into the relationship between workload and effects remains limited. Importantly, establishing a minimum threshold in terms of relative workload would be of relevance for measuring resilience in less well-trained or diseased individuals.

The aim of the present study was to investigate the relationships between exercise intensity and the kinetics of different physiological parameters in healthy human volunteers. To this end, we used samples collected in our previous study using an exercise model appropriate to fit recreational cycling volunteers (Kartaram et al., 2019). The exercise intensity was defined relative to the individual W_*max*_ and was either very strenuous (70% W_*max*_, 1 h), moderate (50% W_*max*_, 1 h) or intermittent (55/85% W_*max*_ in blocks of 2 min, 1 h). Deprivation from fluid intake (inducing mild dehydration) was used as an additional stress intervention. Different types of physiological parameters indicative of metabolic activity (e.g., creatinine, bicarbonate), immunological and hematological functionality (e.g., leukocytes, hemoglobin) and intestinal physiology (citrulline, intestinal fatty acid binding protein, and zonulin) were measured. These parameters were assessed in blood samples taken before, during and at multiple timepoints after a single bout of exercise. In this paper, we present a full overview and detailed analysis of the interaction between the kinetic responses of a wide range of physiological parameters measured in the same study. Our data allows us to select parameters that may serve best as biomarkers of health status and resilience in healthy, less-trained or possibly diseased individuals.

## Materials and Methods

### Ethics

The study was registered at the ISRCTN clinical trial registry (isrctn.com) with code ISRCTN13656034, approved by the Medical Ethics Committee of Wageningen University and Research (WUR), Netherlands, and conducted in accordance with the Declaration of Helsinki (Fortaleza, Brazil, 2013). Informed consent was signed after procedures and guidelines were discussed and before data collection started.

### Participants

Fifteen healthy male recreational cyclists were included in this study. The inclusion criteria were: non-smoking healthy males in the age of 20–35 years, a BMI of between 20 and 25 kg/m^2^ and with at least 2 years recreational cycling experience. Individuals with records of allergies, gastro-intestinal and immune diseases were excluded. Other exclusion criteria were chronic alcohol consumption, use of NSAIDs, corticosteroids, or hard drugs, participation in other clinical studies and blood donation 6 weeks prior to the start of the study. Volunteers were instructed not to perform intense physical activity and to refrain from alcohol consumption for two days prior to the test days. Participants were asked to keep similar dietary habits and to note their daily food intake in a dietary log which was discussed every test day. The day prior to each trial was standardized with regard to the use of alcohol, NSAIDs, artificial sweeteners and dietary supplements. Evening meals were provided to all volunteers prior to the test days to standardize food-intake during the test period. To achieve the dehydrated condition the participants were restricted to a maximum intake of 0.5 L fluid on the day prior to the particular test day. They were also requested to keep records of their training activities during the whole study period.

### Study Design

**FIGURE 1:**
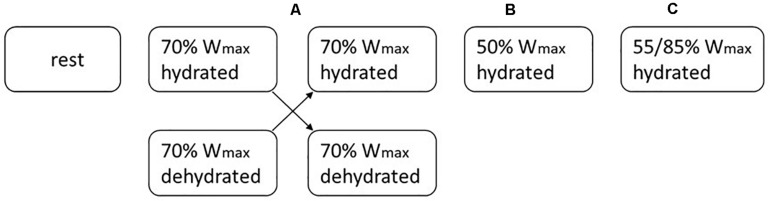
An example of the exercise scheme for one volunteer. Following 1 week of rest, each subject underwent 4 different exercise load interventions which were randomly assigned (blocks **A–C**). The 70% W_*max*_ interventions in hydrated and dehydrated condition (block **A**) were always conducted in sequence. The wash-out-period between the protocols was 1 week.

The study set-up has been described previously (Kartaram et al., 2019). Briefly, participants first underwent a pre-test on an electronically braked cycle ergometer (Lode Excalibur, Groningen, Netherlands) to determine their individual maximal workload (W_*max*_) and maximal oxygen uptake (VO_2max_). The next weeks, they performed a protocol without exercise (P1) and four different exercise protocols (P2–P5) in a block random order. Details on the protocols are given in Table 1. Each participant started with the rest protocol (P1). To enable direct comparison, both high intensity protocols (P2 and P3; at 70% W_*max*_) which only differed in hydration status were always performed sequentially in a random cross-over manner (Block A). Between the trials there was a wash-out period of one week. Figure 1 provides an example of an individual trial sequence. The randomization scheme of the exercise protocols for all volunteers is included as Supplementary Material (Supplement 1).

**TABLE 1:** Experimental protocols with corresponding protocol numbers and exercise intensity.

**Protocol**	**Experimental condition**
P1	Rest condition without exercise
P2	60 min high intensity exercise at 70% W_*max*_
P3	60 min high intensity exercise at 70% W_*max*_, in dehydrated condition
P4	60 min moderate intensity exercise at 50% W_*max*_
P5	60 min intermittent high intensity exercise in blocks of 2 min at 55/85% W_*max*_

The sample size was calculated using the following formula: *N* = (Zα + Zβ)2 x (σ2/δ2). With α = 0.05 (*z*-score 1.96), β = 0.20 (*z*-score 0.84) and σ and δ being the standard deviation and expected difference of the primary outcome measure (intestinal permeability) (Pals et al., 1997; van Wijck et al., 2011; JanssenDuijghuijsen et al., 2016).

### Experimental Procedures

On each test day, a baseline blood sample was obtained following overnight fasting. Before the interventions (exercise or rest) participants received a light breakfast.

Blood samples were taken at different timepoints (displayed as T in hours after the start of exercise), i.e., before (T0), during (T0.5), and at the end (T1) of the exercise period, and at several timepoints post-exercise (T1.5, T2, T3, T6, T24). Samples were collected in Ethylene Diamine Tetra Acetic acid (EDTA) plasma and serum separator tubes (Vacutainer; Becton Dickinson, Breda, Netherlands). EDTA tubes were directly centrifuged at 2000 *g* for 10 min at 4°C. The serum separator tubes were set aside in the dark for at least 30 min at room temperature, after which the tubes were centrifuged at 2000 *g* for 10 min at room temperature. Following a second overnight fasting, the participants arrived the next morning at the laboratory for a final blood collection at 24 h. The obtained plasma and serum were directly aliquoted and stored at −80°C until further analysis.

Participants were asked to rate their perceived exertion on the Borg’ Rating of Perceived Exertion (RPE) scale of 6–20, every 15 min during the one hour exercise and immediately after exercise. They were asked to use any number on the scale to rate their overall experienced effort. A rating of 6 was associated with no exertion at all and a rating of 20 was considered to represent maximal exertion and therefore associated with the most stressful exercise (Borg, 1970). Fluid loss during exercise was compensated with 3 mL tapwater/kg body weight (BW) every 15 min, except during the dehydrated condition (P3). Body weight was measured before and after exercise, to assess changes in hydration status (Table 2). In the post exercise as well as in the post rest period in the rest condition, the participants drank 200 mL of tap water every hour. An extensive lunch was offered after the last blood sampling 5 h post-exercise, after which the participants left with their evening meal. They were asked to fast overnight from 10.00 p.m. and to return to the laboratory at 7.30 a.m. next morning for the 24 h post exercise blood sample, which was drawn by venepuncture.

**TABLE 2:** Mean body weight (BW) ± SD of all subjects per protocol, before and after exercise with the percentage of BW loss.

**Protocol**	**Mean BW pre-exercise (kg)**	**Mean BW post-exercise (kg)**	**BW loss during exercise (kg)**	**% BW loss (%)**
P1	74.8 ± 6.6	75.0 ± 6.6	0.2 ± 0.2	0.3
P2	74.6 ± 6.8	74.4 ± 6.6	−0.3 ± 0.4	−0.4
P3	74.7 ± 6.8	73.6 ± 6.6	−1.1 ± 0.2	−1.4
P4	74.7 ± 6.5	74.7 ± 6.4	0.0 ± 0.2	0.0
P5	74.7 ± 6.8	74.4 ± 6.8	−0.3 ± 0.2	−0.5

### Blood Analysis

We analyzed different domains of markers: hematology (e.g., leukocytes), muscle physiology (e.g., bicarbonate, creatinine), metabolic activity (e.g., glucose, insulin) and intestinal function (e.g., zonulin, intestinal fatty acid binding protein). A list of the parameters in the heatmap (Figure 3), including abbreviations, is provided as Supplementary Material (Supplement 2). Hematologic markers were analyzed using Advia 1200 (Siemens). Other markers were measured using the Cobas 6000 (Roche) (Star-shl, Etten-Leur, Netherlands), according to standard procedures. Zonulin concentrations in serum were determined using an ELISA kit (Immundiagnostik AG, Bensheim, Germany) and measured with an ELISA plate reader at 450 nm against 620 nm as reference (Wegh et al., 2019). Serum intestinal fatty acid binding protein (iFABP) levels were measured using a commercial human ELISA Test Kit (HK406, Hycult Biotech, Uden, Netherlands) according to the manufacturer’s instructions, and analyzed with a multi-detector microplate reader VICTOR^TM^ X3 (PerkinElmer) using Workout v2.5 software.

### Statistical Analysis and Data

Data was analyzed using a multilevel mixed effects model as described previously by Kartaram et al. (2019). This model included terms that capture the random variation between the subjects, between the five experimental protocols per volunteer and also within these experimental protocols. The analysis determined the effects of overall protocol differences, differences between the timepoints within a protocol and the protocol-by-time interaction. The analyses were performed using the statistical software GenStat (version 18) and R (version 3.6.1) (Team R Core, 2013) packages lme4 (Bates et al., 2015) and nIme (Pinheiro et al., 2018). Graphs were created with ggplot2 (R Core Team, 2018). Prior to analysis, the data were log transformed to ensure compatibility with the assumption of a constant standard deviation of the observations.

To focus on statistically significant effects, we corrected the raw *p* values for multiple testing (Benjamini and Hochberg, 1995). Outcomes of statistical tests with *p* < 0.05 were considered statistically significant.

## Results

In this study, 15 volunteers were included. One volunteer did not complete the high intensity exercise protocol at 70%W_*max*_ in dehydrated condition due to personal and practical issues. This volunteer, however, was included because data analysis showed no differences in the means. Subjects characteristics are presented in Table 3.

**TABLE 3:** Baseline characteristics and performance data of the 15 subjects given in mean ± SD.

Age (years)	24.3 ± 2.4
BMI (kg/m^2^)	22.5 ± 1.5
Weight (kg)	75.8 ± 6.7
Length (cm)	183.4 ± 3.8
VO_2__*max*_ (mL/kg/min)	56.9 ± 3.9
W_*max*_ (W)	335.1 ± 39.9

During and following exercise, the kinetics of responses in the volunteers showed intensity-dependent changes in parameters that reflect different physiological domains, e.g., intestinal, metabolic, and hematological.

### Rating of Perceived Exertion and Hydration Status

**FIGURE 2:**
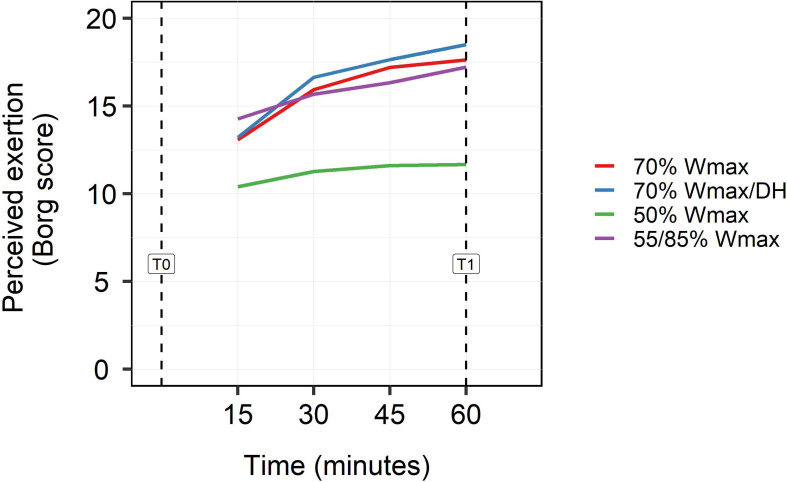
Subjects rating for perceived exertion (RPE) on the Borg scale (0–20) for every 15 min during exercise (15, 30, 45 min) and at the end of exercise (60 min). Perceived exertion was rated for all exercise protocols: high intensity exercise at 70% W_*max*_ in hydrated and dehydrated condition, intermittent high intensity exercise in blocks of 2 min at 55/85% W_*max*_ and moderate exercise at 50% W_*max*_. Statistical analysis showed significant overall effects of timepoints and protocols on rpe (*p* < 0.001) as well as a significant interaction (*p* < 0.001).

Whereas the moderate exercise protocol of 50% W_*max*_ was rated as light (10) to somewhat hard (12) at the end of exercise, the high intensity exercise protocols of 70% and 55/85% W_*max*_ were rated as hard (16) after 30 min cycling and as very hard (20) at the end (Figure 2). The high intensity protocol in dehydrated condition was rated as most exhausting (18–19). Statistical analysis showed significant overall effects of timepoints and protocols on RPE (*p* < 0.001) as well as a significant interaction (*p* < 0.001).

Data on BW (Table 2) showed that BW loss was over three times higher (3.5×) in the dehydrated 70% W_*max*_ protocol (P3) than in the hydrated 70% W_*max*_ protocol (P2). In the dehydrated protocol, the volunteers did not drink and were not compensated for fluid loss during exercise.

### Comparison of Parameters Between Protocols

**FIGURE 3:**
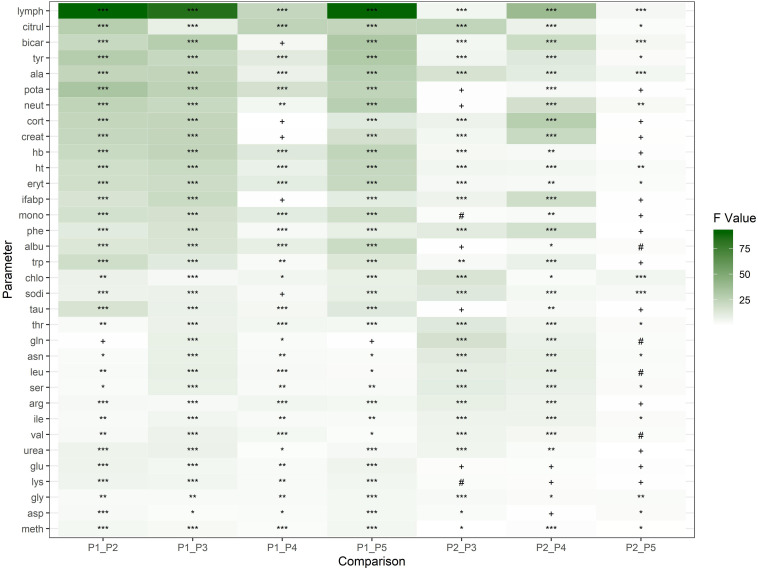
Heatmap representing effect sizes of 34 parameters as calculated by the multilevel mixed effects model. The parameters are shown in rows. All exercise protocols (P2–P5) are compared to the rest condition (P1) and to each other. The protocol effects of these comparisons are represented as contrasts (*F* values 0–100) in columns by green-shaded blocks. Significant *p* values are depicted by asterisks (*): *p < 0.05, **p < 0.01, ***p < 0.001. The *p* values in the range of 0.05–0.1 (#) and 0.01–1 (+) are considered to be not statistically significant.

In order to get an overview of statistically significant differences between protocols and sample timepoints, we compared the values of the measured parameters between the rest protocol (P1) and all other exercise protocols (P2–P5) as well as between the exercise protocols mutually. The levels of the measured parameters of all timepoints were compared to the level of timepoint T0 of the rest protocol (P1). The heatmap showing multilevel ANOVA F values scaled in green shaded blocks is depicted in Figure 3. Darker green colors (higher contrast) represent a higher *F* value (0–100). The significance for a marker (0 < *p* < 0.05) is depicted with asterisks (^∗^): (^∗^*p* < 0.05, ^∗∗^*p* < 0.01, ^∗∗∗^*p* < 0.001). The symbols # and + are considered not to be statistically significant (*p* > 0.05). Thirty four parameters are shown in rows while the comparison between exercise protocols mutual (P2–P5) and with the rest condition (P1) are shown in columns. Compared to rest condition and moderate exercise, high intensity exercises (P2, P3, and P5) induced the clearest differences in the top 20 of all measured parameters. Striking changes occurred in amounts of leukocytes (lymphocytes, neutrophils, and monocytes), erythrocytes and hematocrit, in markers of intestinal status (i.e., citrulline and intestinal fatty acid binding protein), and also in levels of albumin, creatinine, and cortisol. Nearly all parameters showed significant differences between the high intensity exercise protocols, in particular in dehydrated condition (*p* < 0.001).

### Parameters of Physiological and Metabolic Activity

**FIGURE 4:**
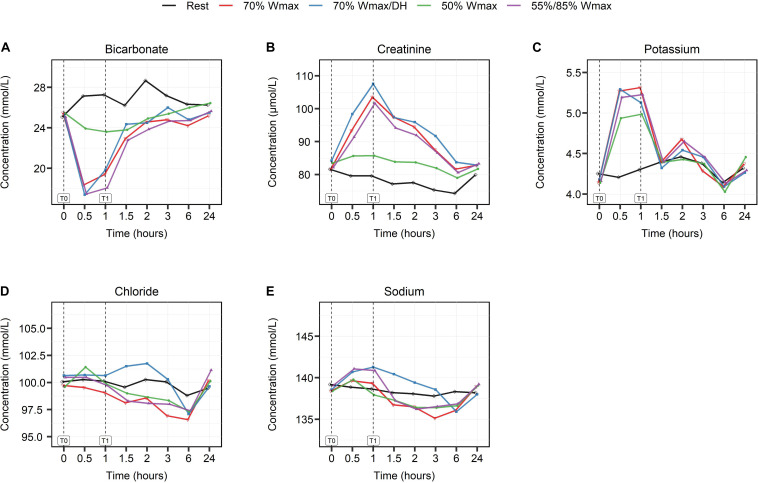
The effect of high- and moderate intensity exercises on markers of physiological and metabolic activity. Bicarbonate **(A)**, creatinine **(B)**, potassium **(C)**, chloride **(D)**, and sodium **(E)** at multiple timepoints shown in hours: at baseline (0), during (0.5), at the end of exercise (1) and post-exercise at different timepoints (1.5, 2, 3, 6, and 24). Serum levels are presented in mmol/L, except for creatinine which is shown in μmol/L. High intensity exercise included 1 h cycling at 70% W_*max*_ in hydrated (red lines) and dehydrated condition (blue lines) and intermittent cycling at 55/85% W_*max*_ in blocks of 2 min (purple lines). Moderate intensity exercise was represented by 1 h cycling at 50% W_*max*_ (green lines). The black lines show serum levels in the rest condition.

Serum bicarbonate (HCO_3_^–^) is considered to be a response marker to acid load. During exercise after only 30 min of exercise (T0.5), HCO_3_^–^ levels declined at all intensities [Figure 4A apart from the moderate (P4), resulted in a maximum decline of HCO_3_^–^ levels from 25 to 17.5 mmol/L (*p* < 0.001)]. Under the moderate intensity exercise condition the HCO_3_^2–^ levels were decreased from 25 to 24 mmol/L at 30 min, but were constantly lower than in the rest condition.

Serum creatinine and potassium (K^+^) levels increased during exercise, also proportional to the intensity of exercise (with *p* < 0.001 and *p* < 0.01 respectively, Figures 4B,C) and regardless of the hydration condition of the subjects.

Serum chloride (Cl^–^) levels (Figure 4D) increased only in the moderate intensity exercise protocol during exercise (*p* < 0.05) while sodium (Na^+^) levels (Figure 4E) increased in all exercise protocols (*p* < 0.01). Na^+^ levels remained increased (T2, *p* < 0.05) only in the high intensity dehydration protocol (P3).

**FIGURE 5:**
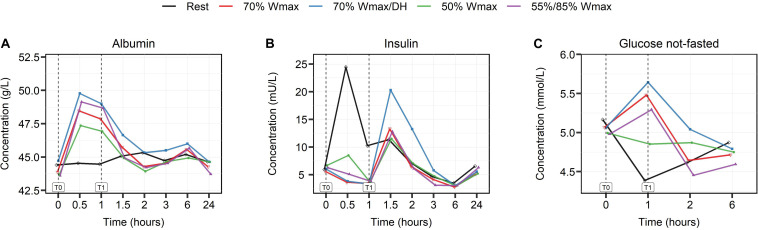
The effect of high- and moderate intensity exercises on serum levels of albumin in g/L **(A)**, insulin in mU/L **(B)**, and plasma glucose levels in mmol/L **(C)**. Levels were measured at multiple timepoints shown in hours: at baseline (0), during (0.5), at the end of exercise (1) and post-exercise at different timepoints (1.5, 2, 3, 6, and 24). High intensity exercise included 1 h cycling at 70% W_*max*_ in hydrated (red lines) and dehydrated condition (blue lines) and intermittent cycling at 55/85% W_*max*_ in blocks of 2 min (purple lines). Moderate intensity exercise was represented by 1 h cycling at 50% W_*max*_ (green lines). The black lines show the serum and plasma levels in the rest condition.

The moderate intensity exercise of 50% W_*max*_ induced the smallest changes in levels of serum albumin (p < 0.001), insulin (p < 0.01) and plasma glucose (p < 0.01), Figures 5A–C, while high intensity exercise of 70% in dehydrated condition induced the largest changes.

### Parameters of Hematological Response

**FIGURE 6:**
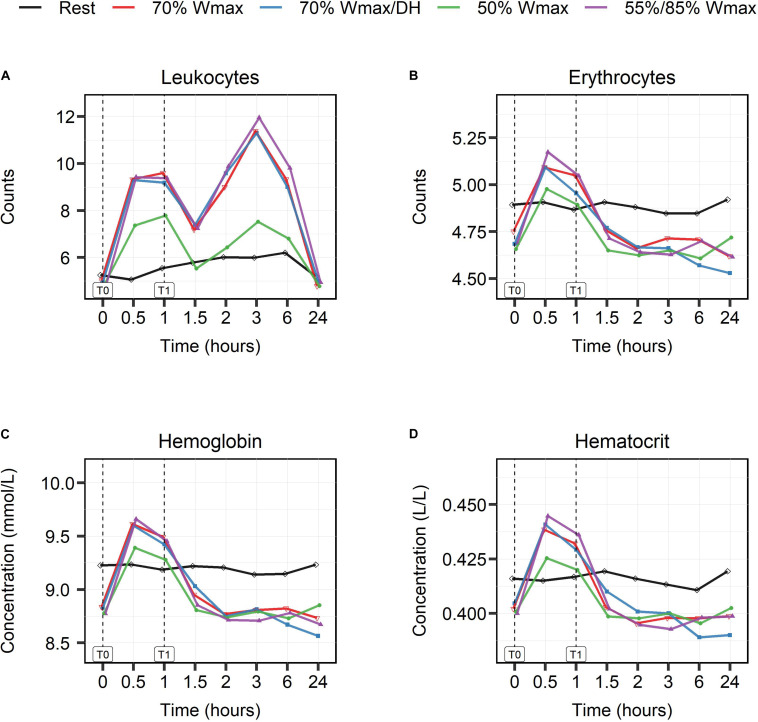
The effect of high- and moderate intensity exercises on markers of hematologic responses. Counts of leukocytes **(A)**, erythrocytes **(B)** per nL, concentrations of hemoglobin **(C)** in mmol/L, and hematocrit **(D)** in L/L were measured at multiple timepoints shown in hours: at baseline (0), during (0.5), at the end of exercise (1) and post-exercise at different timepoints (1.5, 2, 3, 6, and 24). High intensity exercise included 1 h cycling at 70% W_*max*_ in hydrated (red lines) and dehydrated condition (blue lines), and intermittent cycling at 55/85% W_*max*_ in blocks of 2 min (purple lines). Moderate intensity exercise was represented by 1 h cycling at 50% W_*max*_ (green lines). The black lines show the counts and levels in the rest condition.

Leukocyte counts (Figure 6A) showed a biphasic response in all exercise protocols with a first peak at 30 min (T0.5) and a second peak at 3 h (T3). The increase in leukocyte counts was exercise intensity-dependent, and all protocols, except the moderate intensity one, showed comparable kinetics and extent of response. Both peaks in the moderate intensity protocol of 50% W_*max*_ were lower than in the high intensity exercise protocols [(timepoint T0.5, 7/nL vs 9/nL), (timepoint T3, 7/nL vs 12/nL)]. Erythrocyte counts, hemoglobin (Hb) and hematocrit (Ht) levels did not change in the rest condition, but during exercise the levels increased in all exercise protocols (*p* < 0.001, Figures 6B–D) with a peak at 30 min and again the smallest rise in the moderate exercise intensity protocol.

### Parameters of the Intestinal Condition

The levels of parameters of intestinal condition, zonulin, iFABP and citrulline are shown in Table 4 for timepoints T0, T1, T2, and T24. There were no significant differences in zonulin concentrations for the exercise protocols in hydrated condition (P2 and P4), but in dehydrated condition (P3) zonulin concentrations had already increased before exercise (T0) and peaked at T2. Intestinal fatty acid binding protein concentrations were significantly different at the end of exercise (T1, *p* < 0.001) for high intensity exercise in both hydrated (P2) and dehydrated condition (P3). At that timepoint (T1) citrulline showed significant differences (*p* < 0.001) for moderate (P4) and high (P2) intensity exercise in hydrated condition. All significant differences are in comparison with values in the rest condition.

**TABLE 4:** Serum concentrations of zonulin, intestinal fatty acid binding protein (iFABP), and plasma levels of citrulline before (T0), at exercise completion (T1) and post-exercise (T2, T24).

		**T0**	**T1**	**T2**	**T24**
**Zonulin (ng/mL)**	P1	28.3 ± 8.4	31.3 ± 8.8	31.5 ± 4.9	31.8 ± 6.3
	P2	30.6 ± 7.6	34.7 ± 4.8	31.8 ± 3.1	33.9 ± 4.4
	P3	36.7 ± 6.6	**37.1 ± 6.0**	**40.0 ± 4.9**	**36.3 ± 9.6**
	P4	31.5 ± 8.0	33.1 ± 7.4	31.7 ± 7.1	30.6 ± 5.9
**iFABP (pg/mL)**	P1	841.4 ± 448.3	642.7 ± 344.4	415.1 ± 306.2	830.4 ± 455.8
	P2	742.9 ± 341.1	1.262.8 ± 512.7**	788.4 ± 448.4**	817.0 ± 381.1
	P3	689.8 ± 310.0	1.559.8 ± 658.9**	957.0 ± 699.7**	900.5 ± 563.2
	P4	698.8 ± 369.1	598.6 ± 360.3	346.7 ± 164.7	646.5 ± 367.5
**Citrulline (μm/L)**	P1	32.9 ± 6.5	29.0 ± 5.8	41.7 ± 7.3	35.5 ± 6.0
	P2	34.7 ± 5.5	42.6 ± 6.4**	46.2 ± 9.4	37.3 ± 6.5
	P3	36.1 ± 7.4	33.5 ± 8.3	38.5 ± 8.3**	37.4 ± 6.9
	P4	34.9 ± 5.6	43.8 ± 10.2**	50.5 ± 9.8*	36.6 ± 7.0

P1 is rest condition, P2 and P3 are high intensity exercise of 70% W_*max*_ in hydrated and dehydrated condition respectively and P4 is moderate exercise of 50% W_*max*_. The values are given with SD. Significant differences in concentrations are shown with asterisks (^∗^) for *p* values (*p < 0.01, **p < 0.001). Maximum concentrations (peaks) at a given timepoint are shown in bold.

## Discussion

Physical exercise triggers kinetic changes in a range of physiological processes and hence can be used to study processes indicative for homeostatic resilience and general health status. At the same time, more insight is needed in terms of the relationships between exercise workload, interindividual differences and the kinetics of responses. Our results show distinct kinetic responses for several parameters influenced by the extent of exercise. High intensity exercise at 70% W_*max*_ (P2) and intermittent exercise at 55/85% W_*max*_ (P5) had greater impact on many parameters than moderate intensity exercise at 50% W_*max*_ (P4). Strenuous exercise in the mildly dehydrated condition at 70% W_*max*_ (P3) caused specific additional changes, compared to both other forms of strenuous exercise conditions (at 70 and 55/85% W_*max*_). The top 20 parameters showing the greatest differences in all three strenuous exercise conditions (P2, P3, and P5) pointed mostly toward immunological (lymphocytes, neutrophils, monocytes) and intestinal (citrulline, iFABP) responses and toward responses indicative of metabolic activity (amino acids and hormones). Kinetics of citrulline and iFABP as markers of intestinal function have been described previously (Kartaram et al., 2019).

Moderate exercise (P4) particularly affected leukocyte counts, which matches to some extent with previous findings by others that increases in blood leukocyte counts are exercise intensity-dependent (Neves et al., 2015). In this study, the kinetics of leukocyte numbers showed peak responses within half an hour of the start of exercise and also at 2 h post-exercise, suggesting involvement of two different leukocyte subpopulations. Our findings fit to some extent with those of Bessa et al. (2016), but they reported a different order of leukocyte changes, i.e., an early post-exercise increase in neutrophils and followed by an increase in numbers of the lymphocyte subpopulation after 12 h. The difference in kinetics and difference between the time to peak may result from differences in exercise protocols, indicating the importance of the exercise effort on the stress response.

Initially, it was hypothesized that the acute immunological effects of exercise are largely triggered by its effect on the intestinal tract. In particular, by inducing (minor) damage to the epithelial lining, exercise would result in increased leakage of microbial products such as endotoxins into the circulation. In addition, epithelial damage would cause release of immunostimulatory cytokines and chemokines, such as IL6 and IL8. One would therefore expect that iFABP, a marker for intestinal damage (Schellekens et al., 2014; Timmermans et al., 2015) and citrulline, a marker for intestinal metabolic activity (Papadia et al., 2007; Crenn et al., 2008) would show changes prior to the immunological response. Our current data show however, that immunological changes (detected at T0.5) are already manifest prior to the measurable intestinal effects (first detected at T = 1). In a previous study (Kartaram et al., 2019) we have determined the exercise-induced relationship between citrulline and iFABP levels in the context of intestinal function. Citrulline plasma levels were increased most profoundly following moderate exercise intensity, suggesting that at this level of activity, functional enterocyte metabolic capacity is maintained (Crenn et al., 2008). At higher intensity levels, iFABP showed a more pronounced peak, suggesting occurrence of intestinal damage (Kartaram et al., 2019; Table 3). Although we cannot completely exclude this possibility, current results do not support the idea that epithelial damage is causative regarding the observed changes in leukocyte counts.

Volunteers’ perceived exertion during exercise was intensity-dependent. After 30 min cycling at high intensity exercise volunteers scored high on the RPE scale. High intensity exercise in mildly dehydrated condition was perceived to be the most exhaustive. Rating of Perceived Exertion is an accurate predictor for exercise intensity in healthy adults and hence can be used to monitor effectiveness of training. It is also used in persons with medical complaints. For instance, in patients with chronic backpain and COPD, RPE appeared to be a good measurement tool to correlate pain perception with physical exercise (Barker et al., 2003; Barberan-Garcia et al., 2015). In view of future applications of exercise models for health assessments in less-trained or even diseased individuals, it is of interest that we found clear responses for the moderate 50% W_*max*_ exercise as well, which was perceived as being light to somewhat hard. Next, our data shows that levels of many biomarkers had already peaked after 30 min cycling at high intensity exercises (both 70% W_*max*_ exercises). Since 30 min exercise at 70% W_*max*_ was also within the volunteers’ capabilities, though still perceived as relatively high, it might be interesting to apply a 30 min high intensity protocol in future studies.

Our results show that the kinetics of many measured parameters in the mildly dehydrated condition were different from those in the hydrated condition. For example, baseline zonulin levels were already high before the start of exercise indicating impairment of intestinal integrity by dehydration itself. Intestinal permeability may represent decreased functioning of tight junctions. Zonulin is known as a biomarker for intestinal permeability (Fasano, 2011), as was for example also recently confirmed by our group for patients with ulcerative colitis (Wegh et al., 2019). Intestinal fatty acid-binding protein (iFABP, also known as FABP2) is considered a useful biomarker for enterocyte damage (Adriaanse et al., 2013).

Of the various parameters that were analyzed, some provide a more generic picture of the effort levels. For example, the sharp rise of plasma potassium levels, being lower after exercising at 50% W_*max*_ is consistent with findings dating back several decades (Medbø and Sejersted, 1990). Likewise, the sustained increase of Na^+^ plasma levels up to 2 h post-exercise following cycling at 70% W_*max*_ with limited fluid intake (P3) and after intermittent exercise at 55/85% W_*max*_ (P5) most likely reflects dehydration. Another example is the insulin peak during the 1st hour following breakfast when the participants kept rest, which was followed by a second peak that was larger after the heaviest effort (P3). Although such changes can be thought of as normal physiology, their value lies in the fact that their kinetic changes collectively reflect the response to the exercise challenge.

In summary, our results underline the utility of standardized exercise testing in combination with multiple measurements under dynamic conditions. Biomarkers measured for different physiological domains, e.g., intestinal, metabolic, and endocrine processes show distinct kinetic profiles and responses to exercise, dependent on workload and other experimental variables.

Further refinements can likely be obtained with even more extensive measurements, for example using metabolic approaches. Our results merit further studies using standardized exercise protocols in other groups, including elderly, less fit or sick persons. We hypothesize that in diseased and less fit individuals the responses will be different from those in healthy fit individuals, for instance with respect to kinetics, e.g., time to peak, the height of the peak or recovery to baseline values. Moreover, multi-laboratory collaborations and deposition of data in open databases are recommended to facilitate comparison and validation.

## Conclusion

In this study, we demonstrate that high (70 and 55/85% W_*max*_) and moderate (50% W_*max*_) intensity exercise in a bicycle ergometer test produces different time-dependent changes in a broad range of parameters in healthy young men. These parameters are indicative of metabolic activity, immunological and hematological functionality and intestinal physiology and may be considered biomarkers of homeostatic resilience. Furthermore, this study also shows that a relatively small dehydration in healthy fit young men intensifies these time-related changes. Moderate intensity exercise of 50% W_*max*_ reveals sufficient physiological and immunological responses in the bicycle ergometer test and can be employed to test the health condition of less fit individuals.

## Data Availability Statement

The data generated in this study, the graphs included in this publication and part of the statistical analyses are available as an R-package at https://github.com/uashogeschoolutrecht/kinetics. An overview of the data is also provided as Supplementary Material (Supplement 3).

## Ethics Statement

This study is approved by the Medical Ethics Committee of Wageningen University and Research (WUR), Netherlands, and is registered at isrctn.com with code ISRCTN13656034. The patients/participants provided their written informed consent to participate in this study.

## Author Contributions

SK, KN, MM, LM’R, MV, HW, RW, and RP contributed to the conception and design of the experiments. SK, MM, and KN contributed to the study conduction. MV, KM, and IB contributed to the laboratory analysis. ES, MT, SK, and RP contributed to the data and statistical analysis. SK, KN, RP, MT, MV, RW, MM, and JG contributed to the interpretation of data. SK, KN, MM, RP, RW, and JG contributed to the preparation of the manuscript. SK, MT, KN, and RP contributed to the approval of final version. All authors contributed to the revision and editing of the manuscript.

## Conflict of Interest

The authors declare that the research was conducted in the absence of any commercial or financial relationships that could be construed as a potential conflict of interest.
