# Supplementary material for: Kinetics of Physiological Responses as a Measure of Intensity and Hydration Status During Experimental Physical Stress in Human Volunteers
Source: Front Physiol. 2020 Sep 4;11:1006. doi: 10.3389/fphys.2020.01006 (PMC7498705; doi:10.3389/fphys.2020.01006)
Supplement: Supplementary file 1 [file Data_Sheet_1.PDF]

Arbeitsanleitung / Manual

IDK® Zonulin ELISA

Zur in-vitro-Bestimmung von Zonulin-Familien-Peptiden (ZFP) in Serum

For the in vitro determination of zonulin family peptides (ZFP) in serum

Gültig ab / Valid from 2019-05-07

REF **K 5601** Σ 96

REF **K 5601.20** Σ 20 x 96

+2 °C – +8 °C

IVD CE

Immundiagnostik AG, Stubenwald-Allee 8a, 64625 Bensheim, Germany
Tel.: +49 6251 70190-0 Fax: + 49 6251 849430
e.mail: info@immundiagnostik.com www.immundiagnostik.com

Inhalt

1. VERWENDUNGSZWECK 2

2. EINLEITUNG 2

3. INHALT DER TESTPACKUNG 3

4. ERFORDERLICHE LABORGERÄTE UND HILFSMITTEL 4

5. VORBEREITUNG UND LAGERUNG DER REAGENZIEN 4

6. PROBENLAGERUNG UND -VORBEREITUNG 5

Probenlagerung 5
Probenverdünnung 5

7. TESTVORBEREITUNG 5

Vorbereitung der Standards, Kontrollen und Proben 6

8. TESTDURCHFÜHRUNG 6

Testprinzip 6
Pipettierschema 6

9. ERGEBNISSE 7

10. EINSCHRÄNKUNGEN 8

11. QUALITÄTSKONTROLLE 9

Referenzwerte 9

12. TESTCHARAKTERISTIKA 9

Genauigkeit – Präzision 9
Genauigkeit – Richtigkeit 10
Linearität 10
Analytische Sensitivität 11
Analytische Spezifität 11

13. VORSICHTSMASSNAHMEN 11

14. TECHNISCHE MERKMALE 12

15. ALLGEMEINE HINWEISE ZUM TEST 12

16. LITERATUR 13

1. VERWENDUNGSZWECK

Der hier beschriebene Assay ist für die Bestimmung von Zonulin-Familien-Peptiden (ZFP) in Serum geeignet. Nur zur *in-vitro*-Diagnostik.

2. EINLEITUNG

Zonulin ist ein humanes Protein ähnlich dem Zonula-occludens-Toxin von *Vibrio cholerae*, das an der Regulation der interzellulären Kontakte (*tight junctions*) in der Darmwand beteiligt ist. Zonulin bindet an einen spezifischen Rezeptor an der Oberfläche der Epithelzellen der Darmbarriere und aktiviert eine Kaskade biochemischer Ereignisse, welche die Öffnung der *tight junctions* induzieren und als Folge die Durchlässigkeit der Darmepithelzellen erhöhen, so dass verschiedene Substanzen die Darmbarriere passieren und Autoimmunreaktionen auslösen können.

Die Arbeitsgruppe um Fasano hat festgestellt, dass bei Zöliakie- und Typ-1-Diabetes-mellitus-Patienten das Zonulin-System stärker aktiviert ist. Patienten mit aktiver Zöliakie zeigen erhöhte Konzentrationen von Zonulin und Zonulin-Antikörpern im Vergleich zu Nicht-Zöliakiepatienten und Patienten in Remission unter glutenfreier Diät.

Eine erhöhte Darmpermeabilität, die auch unter dem Begriff „*Leaky Gut Syndrom*“ bekannt ist, wird heutzutage mit dem metabolischen Syndrom, Fettleibigkeit und verschiedenen Autoimmun-, Entzündungs- und Tumorerkrankungen in Verbindung gebracht. Außerdem spielt die erhöhte Darmpermeabilität auch bei Krankheiten wie Multipler Sklerose, rheumatoider Arthritis, Asthma und entzündlichen Darmerkrankungen eine bedeutende Rolle.

Der in diesem ELISA verwendete polyklonale Antikörper basiert auf der Zonulinsequenz, die von Wang (Journal of Cell Science, 2000) und di Pierro (Journal of Biological Chemistry, 2001) publiziert wurde.

Die Messwerte des ***IDK***® Zonulin ELISA, der Zonulin-Familien-Peptide nachweist, korrelieren – wie bereits in vielen Veröffentlichungen festgestellt – mit etablierten metabolischen Merkmalen, die mit einer erhöhten Darmpermeabilität wie Insulinresistenz und Adipositas zusammenhängen.

3. INHALT DER TESTPACKUNG

Art.-Nr.	Bezeichnung	Kit-Komponenten	Menge für Art.-Nr.	
			K 5601	K 5601.20
K 5601	PLATE	Mikrotitermodul, vorbeschichtet	12 x 8 Vertiefungen	20 x 12 x 8 Vertiefungen
K 0001.C.100	WASHBUF	Waschpufferkonzentrat, 10x	2 x 100 ml	40 x 100 ml
K 5601	DIL	Verdünnungspuffer, gebrauchsfertig	1 x 100 ml	20 x 100 ml
K 5601	TRACER	Tracerkonzentrat (biotinyliertes ZFP)	1 x 300 µl	20 x 300 µl
K 5601	CONJ	Konjugatkonzentrat, peroxidasemarkiertes Streptavidin	1 x 200 µl	20 x 200 µl
K 5601	STD	Standards, lyophilisiert (Konzentrationen der Spezifikation entnehmen)	4 x 5 vials	25 x 5 vials
K 5601	CTRL 1	Kontrolle, lyophilisiert (Bereich der Spezifi kation entnehmen)	4 x 1 vial	25 x 1 vial
K 5601	CTRL 2	Kontrolle, lyophilisiert (Bereich der Spezifikation entnehmen)	4 x 1 vial	25 x 1 vial
K 0002.15	SUB	Substrat (Tetramethylbenzidin), gebrauchsfertig	1 x 15 ml	20 x 15 ml
K 0003.15	STOP	Stopplösung, gebrauchsfertig	1 x 15 ml	20 x 15 ml

Für Nachbestellungen von Einzelkomponenten verwenden Sie als Bestellnummer die Artikelnummer gefolgt von der Bezeichnung.

4. ERFORDERLICHE LABORGERÄTE UND HILFSMITTEL

- Reinstwasser*
- Präzisionspipetten und Pipettenspitzen für den Einmalgebrauch mit variablen Volumina von 10–1000 µl
- Folie zum Abkleben der Mikrotiterplatte
- Mikrotiterplattenschüttler
- Multikanal- bzw. Multipipette
- Vortex-Mixer
- Laborübliche Glas- oder Plastikröhrchen (Einmalartikel) aus Polypropylen
- Mikrotiterplattenphotometer (benötigte Filter siehe Kapitel 7)

* Immundiagnostik AG empfiehlt die Verwendung von Reinstwasser nach ISO 3696. Es handelt sich dabei um Wasser des Typs 1, welches frei von ungelösten und kolloidalen Ionen und organischen Molekülen ist (frei von Partikeln > 0,2 µm) mit einer elektrischen Leitfähigkeit von 0,055 µS/cm bei 25 °C (≥ 18,2 MΩ cm).

5. VORBEREITUNG UND LAGERUNG DER REAGENZIEN

- Bitte achten Sie bei mehrfachem Einsatz des Kits darauf, dass die Reagenzien wie auf dem Etikett angegeben gelagert und **nur die für den jeweiligen Ansatz benötigten Reagenzienmengen frisch angesetzt werden**. Der Kit kann so bis zu 4 x je nach Probenaufkommen bis zum angegebenen Haltbarkeitsdatum verwendet werden.

- Reagenzien mit einem **Volumen kleiner 100 µl** sollten vor Gebrauch kurz anzentrifugiert werden, um Volumenverluste zu vermeiden.

- **Vorbereitung des Waschpuffers:** Das **Waschpufferkonzentrat (WASHBUF)** muss vor Gebrauch **1:10** in Reinstwasser verdünnt werden (100 ml WASHBUF + 900 ml Reinstwasser), gut mischen. Aufgrund des hohen Salzgehalts im Konzentrat kann es zu Kristallbildungen kommen. Die Kristalle lösen sich bei Raumtemperatur bzw. im Wasserbad bei 37 °C auf. Das **WASHBUF** kann bei **2–8 °C** bis zum angegebenen Haltbarkeitsdatum aufbewahrt werden. Der **Waschpuffer** (1:10 verdünntes WASHBUF) ist **1 Monat bei 2–8 °C** in einem geschlossenen Gefäß haltbar.

- **Die lyophilisierten Standards (STD)** und **Kontrollen (CTRL)** sind bei **2–8 °C** bis zum angegebenen Haltbarkeitsdatum verwendbar. Die Rekonstitutionsvorgaben für STD und CTRL sind dem Spezifikationsdatenblatt zu entnehmen. **Standards und Kontrollen (**rekonstituierte STD und CTRL**) sind nicht stabil und können nicht gelagert werden.**

- **Vorbereitung des Tracers:** Das **Tracer-Konzentrat (TRACER)** wird unmittelbar vor Gebrauch **1:101 in Verdünnungspuffer (DIL)** verdünnt (z. B. 150 µl TRACER + 15 ml DIL). Unverdünntes TRACER ist bei **2–8 °C** bis zum angegebenen Haltbarkeitsdatum stabil. **Tracer** (1:101 verdünntes TRACER) **ist nicht stabil und kann nicht aufbewahrt werden.**
- **Vorbereitung des Konjugats:** Das **Konjugatkonzentrat (CONJ)** wird vor Gebrauch **1:101** in **Verdünnungspuffer (DIL)** verdünnt (z. B. 100 µl CONJ + 10 ml DIL). Das CONJ ist bei **2–8 °C** bis zum angegebenen Haltbarkeitsdatum stabil. **Konjugat** (1:101 verdünntes CONJ) **ist nicht stabil und kann nicht aufbewahrt werden.**
- Alle anderen Testreagenzien sind gebrauchsfertig und, bei **2–8 °C** gelagert, bis zum angegebenen Verfallsdatum (siehe Etikett) verwendbar.

6. PROBENLAGERUNG UND -VORBEREITUNG

Probenlagerung

ZFP ist in unverdünntem Serum für 12 Monate bei -80 °C sowie für 8 Wochen bei -20 °C und für einen Tag bei 2–8 °C stabil. Bei Raumtemperatur ist ZFP nicht stabil.

Probenverdünnung

1.	Je **25 µl Serumprobe** in entsprechend beschriftete Reaktionsgefäße pipettieren.
2.	**475 µl Verdünnungspuffer** zu jeder Probe zugeben. Dies resultiert in einem Verdünnungsfaktor von 1:20.

7. TESTVORBEREITUNG

Im Test dürfen nur **Reagenzien und Proben** verwendet werden, die **Raumtemperatur** (15–30 °C) aufweisen. Vor Gebrauch Reagenzien und Proben gut mischen.

Markieren Sie die Positionen für Standards/Kontrollen/Proben im Protokollblatt.

Die benötigten Mikrotiterstreifen aus dem Kit nehmen. Nicht verwendete Mikrotiterstreifen müssen zusammen mit dem Trockenmittelbeutel in der verschlossenen Aluminiumverpackung bis zum angegebenen Haltbarkeitsdatum bei 2–8 °C gelagert werden.

Wir empfehlen, die Bestimmungen in Doppelwerten durchzuführen.

Vorbereitung der Standards, Kontrollen und Proben

Je **150 µl Standard, Kontrolle** bzw. **verdünnte Probe** in entsprechend beschriftete Reaktionsgefäße pipettieren und mit **150 µl Tracer** versetzen. Gut vortexen und zeitnah im Test einsetzen.

Wichtig:

Führen Sie die Zugabe des Tracers bei den Standards und Kontrollen gleichzeitig mit verdünnten Proben durch, um deren Gleichbehandlung sicherzustellen.

Standards, Kontrollen und Proben sind nun bereit für den Einsatz im Test.

8. TESTDURCHFÜHRUNG

Testprinzip

Dieser ELISA dient zur quantitativen Bestimmung von ZFP im Serum.

Der Test basiert auf der Methode des kompetitiven ELISA. Die zu untersuchenden Proben, Standards und Kontrollen werden mit einem biotinylierten ZFP-Tracer versetzt und anschließend in einer mit einem polyklonalen anti-ZFP-Antikörper beschichteten ELISA-Platte inkubiert. Während der Inkubation kompetitiert das freie Zielantigen in den Proben mit dem biotinylierten ZFP-Tracer um die Bindung der polyklonalen anti-ZFP-Antikörper. Beim zweiten Inkubationsschritt wird peroxidasemarkiertes Streptavidin zugegeben, das an den biotinylierten ZFP-Tracer bindet. Nach einem Waschschritt zur Entfernung ungebundener Komponenten wird das Peroxidasesubstrat Tetramethylbenzidin (TMB) zugegeben. Die Enzymreaktion wird durch Zugabe von Säure abgestoppt. Dadurch erfolgt ein Farbumschlag von blau nach gelb. Die entstandene chromogene Verbindung wird photometrisch bei 450 nm gemessen. Die Intensität der Farbe ist umgekehrt proportional zur Konzentration des gemessenen Analyten, d. h. mit steigender ZFP-Konzentration in der Probe reduziert sich die Konzentration des an den anti-ZFP-Antikörper gebundenen biotinylierten ZFP-Tracers und das Signal nimmt ab. Anhand einer mitgeführten Standardkurve – optische Dichte (Absorption bei 450 nm) versus Standardkonzentration – lässt sich die Konzentration der Probe ermitteln.

Pipettierschema

Im Fall einer automatisierten Abarbeitung des Tests können automatenspezifische Anpassungen der Prozedur notwendig sein, um den jeweiligen technischen Gegebenheiten gerecht zu werden. Für Unterstützung und Rückfragen wenden Sie sich bitte an Ihren Anbieter oder Immundiagnostik AG.

1.	**100 µl der vorbereiteten Standards/Kontrollen/Proben** in die jeweiligen Vertiefungen pipettieren.
2.	Streifen abdecken und **1 Stunde** bei Raumtemperatur (15–30 °C) **unter Schütteln** bei 550 Upm mit einem Orbit von 2 mm inkubieren.
3.	Inhalt der Vertiefungen verwerfen und **5 x mit je 250 µl Waschpuffer** waschen. Nach dem letzten Waschschritt Reste von Waschpuffer durch Ausklopfen auf saugfähigem Papier entfernen.
4.	**100 µl Konjugat** (verdünntes CONJ) in jede Vertiefung pipettieren.
5.	Streifen abdecken und **1 Stunde** bei Raumtemperatur (15–30 °C) **unter Schütteln** bei 550 Upm mit einem Orbit von 2 mm inkubieren.
6.	Inhalt der Vertiefungen verwerfen und **5 x mit je 250 µl Waschpuffer** waschen. Nach dem letzten Waschschritt Reste von Waschpuffer durch Ausklopfen auf saugfähigem Papier entfernen.
7.	**100 µl Substrat** (SUB) in jede Vertiefung pipettieren.
8.	**10–20 Minuten** bei Raumtemperatur (15–30 °C) **im Dunkeln** inkubieren*.
9.	**100 µl Stopplösung** (STOP) in jede Vertiefung pipettieren, gut mischen.
10.	**Extinktion sofort** im Mikrotiterplattenphotometer bei **450 nm** gegen die Referenzwellenlänge 620 nm (oder 690 nm) messen. Ist keine Referenzwellenlänge vorhanden, wird nur bei 450 nm gemessen. Falls die Extinktion einer Probe oder eines Standards den Messbereich des Photometers übersteigt, sollte sofort bei **405 nm** gegen 620 nm (690 nm) gemessen werden.

* Die Intensität der Farbentwicklung ist temperaturabhängig. Es wird empfohlen den Farbumschlag während der Inkubationszeit zu beobachten und entsprechend der Farbentwicklung die Reaktion zu stoppen.

9. ERGEBNISSE

Die unten beschriebenen mathematischen Modelle können alternativ zur Auswertung benutzt werden. Wir empfehlen die 4-Parameter-Funktion:

1. **4-Parameter-Funktion**

   Für die optische Dichte empfehlen wir eine lineare Ordinate und für die Konzentration eine logarithmische Abszisse (bei einer logarithmischen Abszisse muss für den Standard mit der Konzentration 0 ein Wert kleiner 1 eingegeben werden z. B. 0,001).

2. **Punkt-zu-Punkt-Auswertung**

   Für die optische Dichte und für die Konzentration empfehlen wir eine lineare Ordinate bzw. Abszisse.

3. **Gewichtete Spline-Funktion**

   Für die optische Dichte und für die Konzentration empfehlen wir eine lineare Ordinate bzw. Abszisse.

Vor jeder automatischen Auswertung sollte stets eine Kontrolle der Doppelwerte auf Plausibilität („Ausreißerkontrolle“) durchgeführt werden; falls dies nicht durch das verwendete Programm erfolgt, sollte die Kontrolle manuell durchgeführt werden.

Serumproben

Die ermittelten Ergebnisse werden mit dem **Verdünnungsfaktor 20** multipliziert, um die tatsächlichen Konzentrationen zu erhalten.

Sollte ein **anderer Verdünnungsfaktor** verwendet worden sein, so ist die ermittelte Konzentration mit dem verwendeten Verdünnungsfaktor zu multiplizieren.

10. EINSCHRÄNKUNGEN

Proben mit Konzentrationen oberhalb des Messbereichs (Definition siehe unten) können stärker verdünnt und erneut gemessen werden. Bitte beachten Sie diese stärkere Verdünnung bei der Ergebnisberechnung.

Proben mit Konzentrationen unterhalb des Messbereichs (Definition siehe unten) können nicht klar quantifiziert werden.

Die Obergrenze des Messbereichs ergibt sich aus:

> *höchste Konzentration der Standardkurve × anzuwendender Probenverdünnungsfaktor*

Die Untergrenze des Messbereichs ergibt sich aus:

> *LoB × anzuwendender Probenverdünnungsfaktor*

LoB siehe Kapitel „Testcharakteristika“.

11. QUALITÄTSKONTROLLE

Immundiagnostik AG empfiehlt den Einsatz von externen Kontrollen für die interne Qualitätskontrolle, wenn möglich.

Wir empfehlen, bei jedem Testansatz Kontrollen mitzumessen. Die Ergebnisse der Kontrollen müssen auf Richtigkeit überprüft werden. Liegen eine oder mehrere Kontrollen außerhalb des angegebenen Bereiches, kann Immundiagnostik AG die Richtigkeit der Messergebnisse nicht gewährleisten.

Referenzwerte

Anhand einer laborinternen Studie mit Serumproben von augenscheinlich Gesunden (n = 40) wurde ein Medianwert von 34 ng/ml (± 14 ng/ml) ermittelt.

Wir empfehlen jedem Labor, einen eigenen Referenzbereich zu etablieren.

Für Plasmaproben sind eigene Referenzbereiche zu erheben.

12. TESTCHARAKTERISTIKA

Genauigkeit – Präzision

Wiederholbarkeit (Intra-Assay); n=40

Die Wiederholbarkeit wurde mit 2 Serumproben unter gleichbleibenden Bedingungen (Bediener, System, Tag, Kitcharge) bestimmt.

Probe	Mittelwert [ng/ml]	VK [%]
1	43,90	3,5
2	38,38	6,0

Reproduzierbarkeit (Inter-Assay); n=25

Die Reproduzierbarkeit wurde mit 2 Serumproben unter variablen Bedingungen (Bediener, System, Tag, Kitcharge) bestimmt.

Probe	Mittelwert [ng/ml]	VK [%]
1	41,13	7,7
2	46,15	8,3

Genauigkeit – Richtigkeit

Die Richtigkeit gibt das Verhältnis zwischen dem Messergebnis und der wahren Konzentration einer Probe an. 3 Serumproben wurden dafür mit bekannten ZFP-Konzentrationen versetzt und gemessen.

Probe [ng/ml]	Spike [ng/ml]	Erwartet [ng/ml]	Gemessen [ng/ml]	Wiederfindung [%]
10,788	51,58	62,469	66,331	106,2
	37,71	48,494	50,259	103,6
	26,64	37,430	34,469	92,1
12,428	51,58	64,109	70,666	110,2
	37,71	50,134	50,883	101,5
	26,64	39,070	33,032	84,5
13,372	51,58	65,053	70,547	108,4
	37,71	51,078	53,548	104,8
	26,64	40,014	34,266	85,6

Linearität

Die Linearität zeigt die Fähigkeit einer Methode, ein Ergebnis proportional zur Analytkonzentration in einer Probe zu liefern. Sie wurde gemäß CLSI-Richtlinie EP6-A mittels einer seriellen Verdünnung zweier Serumproben nachgewiesen.

Für ZFP in Serum wurde ein lineares Verhalten im Bereich von 3,03 bis 40,25 ng/ml nachgewiesen. Die Nicht-Linearität lag bei weniger als ± 20 %.

Probe	Verdünnung	Erwartet [ng/ml]	Gemessen [ng/ml]	Wiederfindung [%]
A	1:20	36,69	36,69	100,00
	1:40	18,34	19,02	103,70
	1:80	9,17	9,92	108,17
	1:160	4,59	5,91	128,79
B	1:20	40,25	40,25	100,00
	1:40	20,13	20,41	101,41
	1:80	10,06	10,90	108,29
	1:160	5,03	5,64	112,09
	1:320	2,52	3,03	120,52

Analytische Sensitivität

Die im Folgenden aufgeführten Werte wurden in Bezug auf die Standardkurve ohne Berücksichtigung eventuell verwendeter Probenverdünnungsfaktoren ermittelt.

Leerwert (*limit of blank*, LoB)	0,140 ng/ml
Nachweisgrenze (*limit of detection*, LoD)	0,183 ng/ml
Bestimmungsgrenze (*limit of quantitation*, LoQ)	0,183 ng/ml

Die Auswertung wurde gemäß der CLSI-Richtlinie EP17-A2 durchgeführt. Das festgelegte Präzisionsziel für die Bestimmungsgrenze lag bei 20 % VK.

Analytische Spezifität

Die Spezifität wurde nachgewiesen durch Bestimmung der Kreuzreaktivität zu humanem Haptoglobin. Es wurde keine Kreuzreaktivität nachgewiesen.

Getestete Substanz	Eingesetzte Konzentration	Gefundene Konzentration	Fazit
Humanes Haptoglobin	2,9 mg/ml	< 0,02 ng/ml	< LoB

13. VORSICHTSMASSNAHMEN

- Alle im Kit enthaltenen Reagenzien dürfen ausschließlich zur *in-vitro*-Diagnostik verwendet werden.
- Das für Kitkomponenten verwendete humane Material wurde auf HIV, Hepatitis B und Hepatitis C getestet und für negativ befunden. Dennoch wird empfohlen, die Kitkomponenten als Vorsichtsmaßnahme immer wie potentiell infektiöses Material zu behandeln.
- Die Kitkomponenten enthalten zum Schutz vor bakteriellen Kontaminationen Natriumazid oder ProClin. Natriumazid bzw. ProClin sind giftig. Auch Substrate für enzymatische Farbreaktionen sind als giftig und karzinogen beschrieben. Jeder Kontakt mit Haut oder Schleimhaut ist zu vermeiden.
- Die Stopplösung besteht aus verdünnter Schwefelsäure (H_2SO_4). H_2SO_4 ist eine starke Säure und muss auch in verdünnter Form mit Vorsicht benutzt werden. H_2SO_4 verursacht bei Kontakt mit der Haut Verätzungen. Es sollte daher mit Schutzhandschuhen, Schutzkleidung und Schutzbrille gearbeitet werden. Bei Kontakt mit der Säure muss die verätzte Stelle sofort mit viel Wasser gespült werden. Dämpfe nicht einatmen und Inhalation vermeiden.

14. TECHNISCHE MERKMALE

- Reagenzien der Testpackung dürfen nicht mit anderen Chargen gemischt werden. Ferner dürfen Kavitäten unterschiedlicher Mikrotiterplatten, selbst der gleichen Charge, nicht zusammengefügt und zur Analyse verwendet werden.
- Qualitätskontrollen sollten immer mitgemessen werden.
- Die Reagenzien dürfen nach Ablauf des auf der Kitverpackung angegebenen Haltbarkeitsdatums nicht mehr verwendet werden.
- Substratlösung muss vor Gebrauch farblos sein.
- Mikrotiterstreifen müssen während der Inkubationen mit Folie abgedeckt sein.
- Vermeiden Sie Schaumbildung beim Mischen der Reagenzien.
- Stopfen und Verschlüsse verschiedener Reagenzien dürfen nicht vertauscht werden.
- Der Assay ist immer nach der im Kit beigefügten Arbeitsanleitung durchzuführen.

15. ALLGEMEINE HINWEISE ZUM TEST

- Dieser Kit wurde nach der IVD-Richtlinie 98/79/EG hergestellt und in den Verkehr gebracht.
- Für die Qualitätskontrolle sind die für medizinische Laboratorien erstellten Richtlinien zu beachten.
- *IDK*® ist eine Marke der Immundiagnostik AG.
- Die Testcharakteristika wie Inkubationszeiten, Inkubationstemperaturen und Pipettiervolumina der verschiedenen Komponenten wurden vom Hersteller festgelegt. Nicht mit dem Hersteller abgesprochene Veränderungen in der Testdurchführung können die Resultate beeinflussen. Die Firma Immundiagnostik AG übernimmt für die hierdurch entstandenen Schäden und Folgeschäden keine Haftung.
- Bei Gewährleistungsansprüchen ist das beanstandete Material mit schriftlicher Erklärung innerhalb von 14 Tagen zum Hersteller, der Immundiagnostik AG, zurück zu senden.

16. LITERATUR

1. Fasano, A, T Not, W Wang, S Uzzau, I Berti, A Tommasini, and S E Goldblum. 2000. "Zonulin, a Newly Discovered Modulator of Intestinal Permeability, and Its Expression in Coeliac Disease." *Lancet* **355** (9214) (April 29): 1518–9. doi:10.1016/S0140-6736(00)02169-3.
2. Wang, W, S Uzzau, S E Goldblum, and A Fasano. 2000. "Human Zonulin, a Potential Modulator of Intestinal Tight Junctions." *Journal of Cell Science* **113** Pt 24 (December): 4435–40.
3. Fasano, A. 2001. "Intestinal Zonulin: Open Sesame!" *Gut* **49** (2) (August): 159–62.
4. Freemark, Michael, and Lynne L Levitsky. 2003. "Screening for Celiac Disease in Children with Type 1 Diabetes: Two Views of the Controversy." *Diabetes Care* **26** (6) (June): 1932–9.
5. Lazzarotto, Francesca, Daniela Basso, Mario Plebani, Alessandro Moscon, Renato Zanchetta, and Corrado Betterle. 2003. "Celiac Disease and Type 1 Diabetes." *Diabetes Care* **26** (1) (January): 248–9.
6. Watts, Tammara, Irene Berti, Anna Sapone, Tania Gerarduzzi, Tarcisio Not, Ronald Zielke, and Alessio Fasano. 2005. "Role of the Intestinal Tight Junction Modulator Zonulin in the Pathogenesis of Type I Diabetes in BB Diabetic-Prone Rats." *Proceedings of the National Academy of Sciences of the United States of America* **102** (8) (February 22): 2916–21. doi:10.1073/pnas.0500178102.
7. De Magistris, Maria Teresa. 2006. "Zonula Occludens Toxin as a New Promising Adjuvant for Mucosal Vaccines." *Vaccine* **24** Suppl 2 (April 12): S2–60–1.
8. Sapone, Anna, Laura de Magistris, Michelle Pietzak, Maria G Clemente, Amit Tripathi, Francesco Cucca, Rosanna Lampis, et al. 2006. "Zonulin Upregulation Is Associated with Increased Gut Permeability in Subjects with Type 1 Diabetes and Their Relatives." *Diabetes* **55** (5) (May 1): 1443–9. doi:55/5/1443 [pii].
9. Thomas, Karen E, Anna Sapone, Alessio Fasano, and Stefanie N Vogel. 2006. "Gliadin Stimulation of Murine Macrophage Inflammatory Gene Expression and Intestinal Permeability Are MyD88-Dependent: Role of the Innate Immune Response in Celiac Disease." *Journal of Immunology* (Baltimore, Md. : 1950) **176** (4) (February 15): 2512–21.

Verwendete Symbole:

	Temperaturbegrenzung	REF	Bestellnummer
IVD	*In-Vitro*-Diagnostikum	→REF	Zu verwenden mit
	Hersteller	Σ	Inhalt ausreichend für <n> Prüfungen
LOT	Chargenbezeichnung		Verwendbar bis
	Achtung	i	Gebrauchsanweisung beachten
S	Spezifikationsdatenblatt beachten		

Manual

® Zonulin ELISA

For the in vitro determination of zonulin family peptides (ZFP) in serum

Valid from 2019-05-07

REF **K 5601** Σ 96

REF **K 5601.20** Σ 20 x 96

+2 °C +8 °C

IVD CE

Immundiagnostik AG, Stubenwald-Allee 8a, 64625 Bensheim, Germany

Tel.: +49 6251 70190-0 Fax: + 49 6251 849430

e.mail: info@immundiagnostik.com www.immundiagnostik.com

Table of Contents

1. INTENDED USE 17
2. INTRODUCTION 17
3. MATERIAL SUPPLIED 17
4. MATERIAL REQUIRED BUT NOT SUPPLIED 18
5. PREPARATION AND STORAGE OF REAGENTS 19
6. STORAGE AND PREPARATION OF SAMPLES 19
Sample Storage 19
Sample dilution 20
7. PREPARATION OF THE ASSAY 20
Preparation of standards, controls and diluted samples 20
8. ASSAY PROCEDURE 20
Principle of the test 20
Test procedure 21
9. RESULTS 22
10. LIMITATIONS 23
11. QUALITY CONTROL 23
Reference range 23
12. PERFORMANCE CHARACTERISTICS 24
Accuracy – Precision 24
Accuracy – Trueness 24
Linearity 25
Analytical sensitivity 25
Analytical specificity 25
13. PRECAUTIONS 26
14. TECHNICAL HINTS 26
15. GENERAL NOTES ON THE TEST AND TEST PROCEDURE 26
16. REFERENCES 27

1. INTENDED USE

This ELISA is intended for the quantitative determination of zonulin family peptides (ZFP) in serum. For *in vitro* diagnostic use only.

2. INTRODUCTION

Zonulin is a human protein analogue to the zonula occludens toxin derived from *Vibrio cholerae* which regulates tight junctions of the digestive tract. Zonulin binds to a specific receptor on the surface of intestinal epithelia and triggers a cascade of biochemical events which induces tight junction disassembly and a subsequent permeability increase of the intestinal epithelia, allowing some substances to pass through and activate immune reactions.

Fasano and his co-workers found that the zonulin system is more activated in celiac disease and type 1 diabetes mellitus patients. Patients with active celiac disease showed higher levels of zonulin and anti-zonulin antibodies compared to non-celiac patients and patients in remission, who were on a gluten-free diet.

An increased intestinal permeability, also colloquially called 'leaky gut', is nowadays associated with the metabolic syndrome, obesity, and several autoimmune, inflammatory, and neoplastic diseases. Based on evidence, leaky gut plays a meaningful role in diseases such as multiple sclerosis, rheumatoid arthritis, asthma, and inflammatory bowel diseases.

The polyclonal anti-body used in our ELISA is based on the zonulin sequence as published by Wang (Journal of Cell Science, 2000) and di Pierro (Journal of Biological Chemistry, 2001).

Correspondingly, the readings of ***IDK***® Zonulin ELISA detecting zonulin family peptides correlate well - as already found in many papers - with established metabolic traits linked to increased gut permeability, such as insulin resistance and obesity.

3. MATERIAL SUPPLIED

Cat. No.	Label	Kit components	Quantity for cat. no.	
			K 5601	K 5601.20
K 5601	PLATE	Microtiter plate, pre-coated	12 x 8 wells	20 x 12 x 8 wells
K 0001.C.100	WASHBUF	Wash buffer concentrate, 10x	2 x 100 ml	40 x 100 ml

Cat. No.	Label	Kit components	Quantity for cat. no.	
			K 5601	K 5601.20
K 5601	DIL	Dilution buffer, ready-to-use	1 x 100 ml	20 x 100 ml
K 5601	TRACER	Tracer concentrate, biotinylated ZFP	1 x 300 µl	20 x 300 µl
K 5601	CONJ	Conjugate concentrate, peroxidase-labelled streptavidin	1 x 200 µl	20 x 200 µl
K 5601	STD	Standards, lyophilised (see specification for concentrations)	4 x 5 vials	25 x 5 vials
K 5601	CTRL1	Control, lyophilised (see specification for range)	4 x 1 vial	25 x 1 vial
K 5601	CTRL2	Control, ready-to-use (see specification for range)	4 x 1 vial	25 x 1 vial
K 0002.15	SUB	Substrate (tetramethylbenzidine), ready-to-use	1 x 15 ml	20 x 15 ml
K 0003.15	STOP	Stop solution, ready-to-use	1 x 15 ml	20 x 15 ml

For reorders of single components, use the catalogue number followed by the label as product number.

4. MATERIAL REQUIRED BUT NOT SUPPLIED

- Ultrapure water*
- Calibrated precision pipettors and 10–1000 µl single-use tips
- Foil to cover the microtiter plate
- Horizontal microtiter plate shaker
- Multi-channel pipets or repeater pipets
- Vortex
- Standard single-use laboratory glass or plastic vials, cups, etc.
- Microtiter plate reader (required filters see chapter 7)

* Immundiagnostik AG recommends the use of ultrapure water (water type 1; ISO 3696), which is free of undissolved and colloidal ions and organic molecules (free of particles > 0.2 µm) with an electrical conductivity of 0.055 µS/cm at 25 °C (≥ 18.2 MΩ cm).

5. PREPARATION AND STORAGE OF REAGENTS

- To run the assay more than once, ensure that reagents are stored at the conditions stated on the label. **Prepare only the appropriate amount necessary for each run.** The kit can be used up to 4 times within the expiry date stated on the label.
- Reagents with a **volume less than 100 µl** should be centrifuged before use to avoid loss of volume.
- **Preparation of the wash buffer:** The **wash buffer concentrate (WASHBUF)** has to be diluted with ultrapure water **1:10** before use (100 ml WASHBUF + 900 ml ultrapure water), mix well. Crystals could occur due to high salt concentration in the concentrate. Before dilution, the crystals have to be redissolved at room temperature or in a water bath at 37 °C. The **WASHBUF** is stable at **2–8 °C** until the expiry date stated on the label. **Wash buffer** (1:10 diluted WASHBUF) can be stored in a closed flask at **2–8 °C for 1 month**.
- The **lyophilised standards (STD)** and **controls (CTRL)** are stable at **2–8 °C** until the expiry date stated on the label. **Reconstitution** details are given in the **specification data sheet**. **Standards and controls** (reconstituted STD and CTRL) **are not stable and cannot be stored.**
- **Preparation of the tracer:** The **tracer concentrate (TRACER)** has to be diluted **1:101 in dilution buffer** (e.g. 150 µl TRACER + 15 ml DIL) immediately before use. The TRACER is stable at **2–8 °C** until expiry date given on the label. **Tracer** (1:101 diluted TRACER) **is not stable and cannot be stored.**
- **Preparation of the conjugate:** Immediately before use, the **conjugate concentrate (CONJ)** has to be diluted **1:101** in dilution buffer (e.g. 100 µl CONJ + 10 ml DIL). The CONJ is stable at **2–8 °C** until expiry date stated on the label. **Conjugate** (1:101 diluted CONJ) **is not stable and cannot be stored.**
- All other test reagents are ready-to-use. Test reagents are stable until the expiry date (see label) when stored at **2–8 °C**.

6. STORAGE AND PREPARATION OF SAMPLES

Sample Storage

ZFP is stable in undiluted serum for 12 months at -80 °C as well as for 8 weeks at -20 °C and for 1 day at 2–8 °C. ZFP is not stable at room temperature.

Sample dilution

1.	Pipet each **25 µl** of **serum samples** in the respectively labelled reaction tubes.
2.	Add **475 µl** of **dilution buffer** to each sample. This results in a dilution factor of 20.

7. PREPARATION OF THE ASSAY

Prior to use, allow all reagents and samples to come to room temperature (15–30 °C) and mix well.

Mark the positions of standards/controls/samples on a protocol sheet.

Take as many microtiter strips as needed from the kit. Store unused strips together with the desiccant bag in the closed aluminium packaging at 2–8 ° C. Strips are stable until expiry date stated on the label.

We recommend to carry out the tests in duplicate.

Preparation of standards, controls and diluted samples

Transfer **150 µl** of each **standard**, **control** or **diluted sample** in the correspondingly labelled reaction tubes and add **150 µl** of **tracer.** Vortex well and use promptly in the test.

Important:

Carry out the addition of tracer simultaneously with standards, controls and diluted samples in order to ensure equal treatment.

Standards, controls and samples are now ready for use in the test.

8. ASSAY PROCEDURE

Principle of the test

This ELISA is designed for the quantitative determination of ZFP in serum samples.

This assay is based on the method of competitive ELISA. As a first preparation step, a biotinylated ZFP tracer is added to the samples, standards and controls. Afterwards, aliquots of the treated samples, standards and controls are transferred and incubated in microtiter plate wells coated with polyclonal anti-ZFP antibodies. During the incubation, the free target antigen in the samples competes with the biotinylated ZFP tracer for the binding of the polyclonal anti-ZFP antibodies immobilised on the microtiter plate wells. The unbound components are removed by a washing step.

During a second incubation step, peroxidase-labelled streptavidin, which binds to the biotinylated ZFP tracer, is added into each microtiter well. After a washing step to remove the unbound components, the peroxidase substrate tetramethylbenzidine (TMB) is added. Finally, the enzymatic reaction is terminated by an acidic stop solution. The colour changes from blue to yellow and the absorbance is measured in the photometer at 450 nm. The intensity of the yellow colour is inverse proportional to the ZFP concentration in the sample; this means, high ZFP concentration in the sample reduces the concentration of the biotinylated ZFP tracer bound to the immobilised anti-ZFP antibodies and lowers the photometric signal. A dose response curve of absorbance unit (optical density, OD at 450 nm) vs. concentration is generated, using the values obtained from the standard. ZFP, present in the patient samples, is determined directly from this curve.

Test procedure

For automated ELISA processors, the given protocol may need to be adjusted according to the specific features of the respective automated platform. For further details please contact your supplier or Immundiagnostik AG.

1.	Add each **100 µl of the prepared standards/controls/samples** into the respective wells.
2.	Cover the strips and incubate for **1 hour shaking** on a horizontal shaker at 550 rpm with an orbit of 2 mm at room temperature (15–30 °C).
3.	Discard the content of each well and wash **5 times** with **250 µl wash buffer**. After the final washing step, remove residual wash buffer by firmly tapping the plate on absorbent paper.
4.	Add **100 µl conjugate** (diluted CONJ) into each well.
5.	Cover the strips and incubate for **1 hour shaking** on a horizontal shaker at 550 rpm with an orbit of 2 mm at room temperature (15–30 °C).
6.	Discard the content of each well and wash **5 times** with **250 µl wash buffer**. After the final washing step, remove residual wash buffer by firmly tapping the plate on absorbent paper.
7.	Add **100 µl substrate** (SUB) into each well.
8.	Incubate for **10–20 minutes** at room temperature (15–30 °C)* **in the dark**.
9.	Add **100 µl stop solution** (STOP) into each well and mix well.

10.	Determine **absorption immediately** with an ELISA reader at **450 nm** against 620 nm (or 690 nm) as a reference. If no reference wavelength is available, read only at 450 nm. If the extinction of a sample or a standard exceeds the range of the photometer, absorption must be measured immediately at **405 nm** against 620 nm as a reference.

* The intensity of the colour change is temperature sensitive. We recommend observing the colour change and stopping the reaction upon good differentiation.

9. RESULTS

The following algorithms can be used alternatively to calculate the results. We recommend using the 4 parameter algorithm.

1. 4 parameter algorithm

It is recommended to use a linear ordinate for the optical density and a logarithmic abscissa for the concentration. When using a logarithmic abscissa, the zero standard must be specified with a value less than 1 (e. g. 0.001).

2. Point-to-point calculation

We recommend a linear ordinate for the optical density and a linear abscissa for the concentration.

3. Spline algorithm

We recommend a linear ordinate for the optical density and a linear abscissa for the concentration.

The plausibility of the duplicate values should be examined before the automatic evaluation of the results. If this option is not available with the programme used, the duplicate values should be evaluated manually.

Serum samples

The obtained results have to be multiplied by the **dilution factor of 20** to get the actual concentrations.

In case **another dilution factor** has been used, multiply the obtained result by the dilution factor used.

10. LIMITATIONS

Samples with concentrations above the measurement range (see definition below) can be further diluted and re-assayed. Please consider this higher dilution when calculating the results.

Samples with concentrations lower than the measurement (see definition below) range cannot be clearly quantified.

The upper limit of the measurement range can be calculated as:

highest concentration of the standard curve × sample dilution factor to be used

The lower limit of the measurement range can be calculated as:

LoB × sample dilution factor to be used

LoB see chapter "Performance characteristics".

11. QUALITY CONTROL

Immundiagnostik AG recommends the use of external controls for internal quality control, if possible.

Control samples should be analysed with each run. Results, generated from the analysis of control samples, should be evaluated for acceptability using appropriate statistical methods. The results for the patient samples may not be valid if within the same assay one or more values of the quality control sample are outside the acceptable limits.

Reference range

Based on Immundiagnostik AG studies of serum samples of apparently healthy persons (n = 40), a median value of 34 ng/ml (± 14 ng/ml) was estimated.

We recommend each laboratory to establish its own reference range.

Establish own reference ranges for plasma samples.

12. PERFORMANCE CHARACTERISTICS

Accuracy – Precision

Repeatability (Intra-Assay); n=40

The repeatability was assessed with 2 serum samples under constant parameters (same operator, measurement system, day and kit lot).

Sample	Mean value [ng/ml]	CV [%]
1	43.90	3.5
2	38.38	6.0

Reproducibility (Inter-Assay); n=25

The reproducibility was assessed with 2 serum samples under varying parameters (different operators, measurement systems, days and kit lots).

Sample	Mean value [ng/ml]	CV [%]
1	41.13	7.7
2	46.15	8.3

Accuracy – Trueness

The trueness states the closeness of the agreement between the result of a measurement and the true value of the measurand. Therefore, ZFP spikes with known concentrations were added to 3 different serum samples.

Sample [ng/ml]	Spike [ng/ml]	Expected [ng/ml]	Obtained [ng/ml]	Recovery [%]
10.788	51.58	62.469	66.331	106.2
	37.71	48.494	50.259	103.6
	26.64	37.430	34.469	92.1
12.428	51.58	64.109	70.666	110.2
	37.71	50.134	50.883	101.5
	26.64	39.070	33.032	84.5
13.372	51.58	65.053	70.547	108.4
	37.71	51.078	53.548	104.8
	26.64	40.014	34.266	85.6

Linearity

The linearity states the ability of a method to provide results proportional to the concentration of analyte in the test sample within a given range. This was assessed according to CLSI guideline EP6-A with a serial dilution of 2 different serum samples.

For ZFP in serum, the method has been demonstrated to be linear from 3.03–40.25 ng/ml, showing a non-linear behaviour of less than ± 20 % in this interval for concentrations greater than the Limit of Quantitation.

Sample	Dilution	Expected [ng/ml]	Obtained [ng/ml]	Recovery [%]
A	1:20	36.69	36.69	100.00
	1:40	18.34	19.02	103.70
	1:80	9.17	9.92	108.17
	1:160	4.59	5.91	128.79
B	1:20	40.25	40.25	100.00
	1:40	20.13	20.41	101.41
	1:80	10.06	10.90	108.29
	1:160	5.03	5.64	112.09
	1:320	2.52	3.03	120.52

Analytical sensitivity

The following values have been estimated based on the concentrations of the standard without considering possibly used sample dilution factors

Limit of blank, LoB 0.140 ng/ml

Limit of detection, LoD 0.183 ng/ml

Limit of quantitation, LoQ 0.183 ng/ml

The evaluation was performed according to the CLSI guideline EP17-A2. The specified accuracy goal for the LoQ was 20 % CV.

Analytical specificity

The specificity of the antibody was tested by measuring the cross-reactivity against human haptoglobin. There was no cross-reactivity observed.

Substance tested	Concentration added	Concentration obtained	Conclusion
Human haptoglobin	2.9 mg/ml	< 0.02 ng/ml	< LoB

13. PRECAUTIONS

- All reagents in the kit package are for *in vitro* diagnostic use only.
- Human materials used in kit components were tested and found to be negative for HIV, Hepatitis B and Hepatitis C. However, for safety reasons, all kit components should be treated as potentially infectious.
- Kit reagents contain sodium azide or Proclin as bactericides. Sodium azide and Proclin are toxic. Substrates for the enzymatic colour reactions are toxic and carcinogenic. Avoid contact with skin or mucous membranes.
- The stop solution consists of diluted sulphuric acid, a strong acid. Although diluted, it still must be handled with care. It can cause burns and should be handled with gloves, eye protection, and appropriate protective clothing. Any spill should be wiped up immediately with copious quantities of water. Do not breath vapour and avoid inhalation.

14. TECHNICAL HINTS

- Do not interchange different lot numbers of any kit component within the same assay. Furthermore we recommend not assembling wells of different microtiter plates for analysis, even if they are of the same batch.
- Control samples should be analysed with each run.
- Reagents should not be used beyond the expiration date stated on kit label.
- Substrate solution should remain colourless until use.
- To ensure accurate results, proper adhesion of plate sealers during incubation steps is necessary.
- Avoid foaming when mixing reagents.
- Do not mix plugs and caps from different reagents.
- The assay should always be performed according to the enclosed manual.

15. GENERAL NOTES ON THE TEST AND TEST PROCEDURE

- This assay was produced and distributed according to the IVD guidelines of 98/79/EC.
- The guidelines for medical laboratories should be followed.
- *IDK*® is a trademark of Immundiagnostik AG.

- Incubation time, incubation temperature and pipetting volumes of the components are defined by the producer. Any variation of the test procedure, which is not coordinated with the producer, may influence the results of the test. Immundiagnostik AG can therefore not be held responsible for any damage resulting from incorrect use.
- Warranty claims and complaints regarding deficiencies must be logged within 14 days after receipt of the product. The product should be send to Immundiagnostik AG along with a written complaint.

16. REFERENCES

1. Fasano, A, T Not, W Wang, S Uzzau, I Berti, A Tommasini, and S E Goldblum. 2000. "Zonulin, a Newly Discovered Modulator of Intestinal Permeability, and Its Expression in Coeliac Disease." *Lancet* **355** (9214) (April 29): 1518–9. doi:10.1016/S0140-6736(00)02169-3.
2. Wang, W, S Uzzau, S E Goldblum, and A Fasano. 2000. "Human Zonulin, a Potential Modulator of Intestinal Tight Junctions." *Journal of Cell Science* **113** Pt 24 (December): 4435–40.
3. Fasano, A. 2001. "Intestinal Zonulin: Open Sesame!" *Gut* **49** (2) (August): 159–62.
4. Freemark, Michael, and Lynne L Levitsky. 2003. "Screening for Celiac Disease in Children with Type 1 Diabetes: Two Views of the Controversy." *Diabetes Care* **26** (6) (June): 1932–9.
5. Lazzarotto, Francesca, Daniela Basso, Mario Plebani, Alessandro Moscon, Renato Zanchetta, and Corrado Betterle. 2003. "Celiac Disease and Type 1 Diabetes." *Diabetes Care* **26** (1) (January): 248–9.
6. Watts, Tammara, Irene Berti, Anna Sapone, Tania Gerarduzzi, Tarcisio Not, Ronald Zielke, and Alessio Fasano. 2005. "Role of the Intestinal Tight Junction Modulator Zonulin in the Pathogenesis of Type I Diabetes in BB Diabetic-Prone Rats." *Proceedings of the National Academy of Sciences of the United States of America* **102** (8) (February 22): 2916–21. doi:10.1073/pnas.0500178102.
7. De Magistris, Maria Teresa. 2006. "Zonula Occludens Toxin as a New Promising Adjuvant for Mucosal Vaccines." *Vaccine* **24** Suppl 2 (April 12): S2–60–1.
8. Sapone, Anna, Laura de Magistris, Michelle Pietzak, Maria G Clemente, Amit Tripathi, Francesco Cucca, Rosanna Lampis, et al. 2006. "Zonulin Upregulation Is Associated with Increased Gut Permeability in Subjects with Type 1 Diabetes and Their Relatives." *Diabetes* **55** (5) (May 1): 1443–9. doi:55/5/1443 [pii].

9. Thomas, Karen E, Anna Sapone, Alessio Fasano, and Stefanie N Vogel. 2006. "Gliadin Stimulation of Murine Macrophage Inflammatory Gene Expression and Intestinal Permeability Are MyD88-Dependent: Role of the Innate Immune Response in Celiac Disease." *Journal of Immunology* (Baltimore, Md. : 1950) **176** (4) (February 15): 2512–21.

Used symbols:

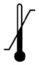	Temperature limitation	REF	Catalogue Number
IVD	In Vitro Diagnostic Medical Device	→REF	To be used with
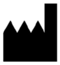	Manufacturer	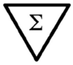	Contains sufficient for <n> tests
LOT	Lot number	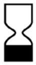	Use by
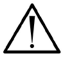	Attention	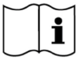	Consult instructions for use
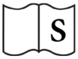	Consult specification data sheet		

Immundiagnostik AG

Stubenwald-Allee 8a
D-64625 Bensheim

Tel.: +49 (0) 62 51/70 19 00
Fax: +49 (0) 62 51/84 94 30

info@immundiagnostik.com
www.immundiagnostik.com
