# Supplementary material for: Kinetics of Physiological Responses as a Measure of Intensity and Hydration Status During Experimental Physical Stress in Human Volunteers
Source: Front Physiol. 2020 Sep 4;11:1006. doi: 10.3389/fphys.2020.01006 (PMC7498705; doi:10.3389/fphys.2020.01006)
Supplement: Supplementary file 2 [file Table_1.pdf]

Supplementary Material

Supplement 1. The randomization scheme of the exercise protocols for all volunteers An individual sequence of exercise protocols was assigned to each volunteer.

**P1**	**P2**	**P3**	**P5**	**P4**
**P1**	P3	P2	P5	P4
**P1**	P5	P2	P3	P4
**P1**	P4	P5	P3	P2
**P1**	P4	P2	P3	P5
**P1**	P5	P4	P3	P2
**P1**	P3	P2	P4	P5
**P1**	P4	P5	P2	P3
**P1**	P5	P3	P2	P4
**P1**	P4	P3	P2	P5
**P1**	P5	P4	P2	P3
**P1**	P2	P3	P5	P4
**P1**	P2	P3	P4	P5
**P1**	P3	P2	P5	P4
**P1**	P5	P4	P2	-
