# Supplementary material for: Kinetics of Physiological Responses as a Measure of Intensity and Hydration Status During Experimental Physical Stress in Human Volunteers
Source: Front Physiol. 2020 Sep 4;11:1006. doi: 10.3389/fphys.2020.01006 (PMC7498705; doi:10.3389/fphys.2020.01006)
Supplement: Supplementary file 3 [file Table_2.pdf]

Supplementary Material

Supplement 2. The abbreviations with the corresponding full names of the 34 parameters which are listed in the heatmap (Figure 3).

Nr	Abbreviation parameter	Full name parameter
1	lymph	lymphocytes
2	citrul	citrulline
3	bicar	bicarbonate
4	tyr	tyrosine
5	ala	alanine
6	pota	potassium
7	neut	neutrophils
8	cort	cortisol
9	creat	creatinine
10	hb	hemoglobin
11	ht	hematocrite
12	eryt	erythrocytes
13	ifabp	intestinal fatty acid binding protein
14	mono	monocytes
15	phe	phenylalanine
16	albu	albumine
17	trp	tryptophan
18	chlo	chloride
19	sodi	sodium
20	tau	taurine
21	thr	threonine
22	gln	glutamine
23	asn	asparagine
24	leu	leucine
25	ser	serine
26	arg	arginine
27	ile	isoleucine
28	val	valine
29	urea	urea
30	glu	glutamic acid
31	lys	lysine
32	gly	glycine
33	asp	asparic acid
34	meth	methionine
