# Supplementary material for: Kinetics of Physiological Responses as a Measure of Intensity and Hydration Status During Experimental Physical Stress in Human Volunteers
Source: Front Physiol. 2020 Sep 4;11:1006. doi: 10.3389/fphys.2020.01006 (PMC7498705; doi:10.3389/fphys.2020.01006)
Supplement: Supplementary file 4 [file Table_3.pdf]

Supplementary Material

Supplement 3. An overview of the data in this publication. The data and the graphs included are also available as an R-package at
https://uashogeschoolutrecht.github.io/kinetics_tables.html

protocol	time	analyte	mean	sd	n_obs
P1	0.0	ala	341.338	80.73	15
P1	0.5	ala	366.255	89.81	15
P1	1.0	ala	386.725	84.74	15
P1	1.5	ala	992.711	308.07	15
P1	2.0	ala	799.408	215.06	14
P1	24.0	ala	393.618	69.22	15
P1	3.0	ala	466.513	125.38	15
P1	6.0	ala	335.779	75.26	15
P2	0.0	ala	357.909	42.42	15
P2	0.5	ala	616.712	46.59	14
P2	1.0	ala	668.840	66.56	15
P2	1.5	ala	1208.113	277.14	15
P2	2.0	ala	900.982	189.79	14
P2	24.0	ala	355.717	69.83	15
P2	3.0	ala	462.063	94.60	15
P2	6.0	ala	320.843	52.00	14
P3	0.0	ala	357.735	72.24	14
P3	0.5	ala	603.730	77.93	13
P3	1.0	ala	604.195	118.97	13
P3	1.5	ala	754.698	182.47	14
P3	2.0	ala	942.173	242.25	12
P3	24.0	ala	375.976	59.46	14
P3	3.0	ala	496.984	140.82	13
P3	6.0	ala	311.157	48.98	12
P4	0.0	ala	377.211	77.99	15
P4	0.5	ala	491.294	115.44	14

protocol	time	analyte	mean	sd	n_obs
P4	1.0	ala	481.333	98.03	15
P4	1.5	ala	1141.309	238.92	15
P4	2.0	ala	758.676	126.61	14
P4	24.0	ala	356.489	52.00	15
P4	3.0	ala	450.945	95.02	14
P4	6.0	ala	311.806	57.92	14
P5	0.0	ala	356.556	85.42	15
P5	0.5	ala	635.664	100.83	15
P5	1.0	ala	660.353	145.20	15
P5	1.5	ala	1238.774	284.32	14
P5	2.0	ala	986.901	234.98	14
P5	24.0	ala	346.989	77.57	15
P5	3.0	ala	456.491	118.24	15
P5	6.0	ala	304.783	64.42	15
P1	0.0	ALB	44.400	1.96	15
P1	0.5	ALB	44.533	2.50	15
P1	1.0	ALB	44.467	3.14	15
P1	1.5	ALB	45.071	2.90	14
P1	2.0	ALB	45.333	2.74	15
P1	24.0	ALB	44.667	2.19	15
P1	3.0	ALB	44.733	2.87	15
P1	6.0	ALB	45.200	2.83	15
P2	0.0	ALB	43.867	1.85	15
P2	0.5	ALB	48.462	2.33	13
P2	1.0	ALB	47.867	2.39	15
P2	1.5	ALB	45.733	2.28	15
P2	2.0	ALB	44.286	2.43	14
P2	24.0	ALB	44.267	2.22	15
P2	3.0	ALB	44.533	2.33	15
P2	6.0	ALB	45.571	2.62	14
P3	0.0	ALB	44.714	2.23	14
P3	0.5	ALB	49.769	2.46	13
P3	1.0	ALB	49.000	2.83	14
P3	1.5	ALB	46.643	2.59	14

protocol	time	analyte	mean	sd	n_obs
P3	2.0	ALB	45.333	2.60	12
P3	24.0	ALB	44.643	2.40	14
P3	3.0	ALB	45.500	2.14	14
P3	6.0	ALB	46.000	2.76	11
P4	0.0	ALB	43.667	2.16	15
P4	0.5	ALB	47.357	2.40	14
P4	1.0	ALB	46.933	1.94	15
P4	1.5	ALB	44.933	2.68	15
P4	2.0	ALB	43.929	1.94	14
P4	24.0	ALB	44.625	1.93	16
P4	3.0	ALB	44.667	1.92	15
P4	6.0	ALB	44.929	2.64	14
P5	0.0	ALB	43.600	1.64	15
P5	0.5	ALB	49.133	1.96	15
P5	1.0	ALB	48.667	2.58	15
P5	1.5	ALB	44.929	1.73	14
P5	2.0	ALB	44.214	2.19	14
P5	24.0	ALB	43.714	2.84	14
P5	3.0	ALB	44.533	2.72	15
P5	6.0	ALB	45.667	2.35	15
P1	0.0	arg	94.777	12.34	15
P1	0.5	arg	116.724	22.02	15
P1	1.0	arg	114.290	13.82	15
P1	1.5	arg	110.013	15.20	15
P1	2.0	arg	109.196	13.85	14
P1	24.0	arg	102.215	11.03	15
P1	3.0	arg	93.574	14.30	15
P1	6.0	arg	86.079	10.84	15
P2	0.0	arg	99.258	10.58	15
P2	0.5	arg	116.921	15.44	14
P2	1.0	arg	123.293	12.56	15
P2	1.5	arg	117.168	15.30	15
P2	2.0	arg	112.362	15.13	14
P2	24.0	arg	95.259	8.33	15

protocol	time	analyte	mean	sd	n_obs
P2	3.0	arg	89.491	10.79	15
P2	6.0	arg	85.446	9.23	14
P3	0.0	arg	99.448	15.13	14
P3	0.5	arg	108.671	13.53	13
P3	1.0	arg	109.286	17.59	13
P3	1.5	arg	113.196	16.69	14
P3	2.0	arg	114.766	17.65	12
P3	24.0	arg	95.241	10.27	14
P3	3.0	arg	96.844	9.17	13
P3	6.0	arg	83.952	8.93	12
P4	0.0	arg	96.891	13.37	15
P4	0.5	arg	129.740	20.38	14
P4	1.0	arg	128.451	16.05	15
P4	1.5	arg	115.941	17.99	15
P4	2.0	arg	109.979	16.58	14
P4	24.0	arg	96.909	10.91	15
P4	3.0	arg	95.002	9.33	14
P4	6.0	arg	87.806	7.75	14
P5	0.0	arg	97.241	10.03	15
P5	0.5	arg	115.469	14.92	15
P5	1.0	arg	120.703	16.27	15
P5	1.5	arg	114.650	14.35	14
P5	2.0	arg	113.706	12.70	14
P5	24.0	arg	92.571	9.78	15
P5	3.0	arg	88.768	11.19	15
P5	6.0	arg	84.219	8.21	15
P1	0.0	asn	50.573	7.76	15
P1	0.5	asn	59.560	10.35	15
P1	1.0	asn	59.236	9.22	15
P1	1.5	asn	76.997	14.73	15
P1	2.0	asn	65.957	13.69	14
P1	24.0	asn	55.401	7.04	15
P1	3.0	asn	49.099	8.88	15
P1	6.0	asn	46.966	7.13	15

protocol	time	analyte	mean	sd	n_obs
P2	0.0	asn	52.814	6.80	15
P2	0.5	asn	56.534	9.50	14
P2	1.0	asn	59.397	10.13	15
P2	1.5	asn	72.675	13.50	15
P2	2.0	asn	65.674	15.04	14
P2	24.0	asn	55.207	5.91	15
P2	3.0	asn	43.841	8.04	15
P2	6.0	asn	45.481	6.56	14
P3	0.0	asn	49.114	8.46	14
P3	0.5	asn	49.409	8.16	13
P3	1.0	asn	51.440	9.08	13
P3	1.5	asn	57.337	10.32	14
P3	2.0	asn	62.197	11.51	12
P3	24.0	asn	54.651	6.79	14
P3	3.0	asn	44.385	10.23	13
P3	6.0	asn	42.151	7.36	12
P4	0.0	asn	51.144	7.25	15
P4	0.5	asn	62.441	11.88	14
P4	1.0	asn	60.943	8.97	15
P4	1.5	asn	73.609	14.43	15
P4	2.0	asn	60.074	12.66	14
P4	24.0	asn	52.297	6.81	15
P4	3.0	asn	46.027	7.41	14
P4	6.0	asn	44.988	6.32	14
P5	0.0	asn	51.724	6.25	15
P5	0.5	asn	55.467	8.71	15
P5	1.0	asn	57.335	8.50	15
P5	1.5	asn	72.266	11.86	14
P5	2.0	asn	67.682	10.48	14
P5	24.0	asn	54.508	6.70	15
P5	3.0	asn	43.197	7.36	15
P5	6.0	asn	44.097	6.63	15
P1	0.0	asp	5.275	1.63	15
P1	0.5	asp	6.070	3.25	15

protocol	time	analyte	mean	sd	n_obs
P1	1.0	asp	5.351	1.90	15
P1	1.5	asp	8.700	2.86	15
P1	2.0	asp	7.145	2.63	14
P1	24.0	asp	6.852	5.39	15
P1	3.0	asp	5.747	1.65	15
P1	6.0	asp	4.785	1.32	15
P2	0.0	asp	5.891	1.84	15
P2	0.5	asp	6.416	1.24	14
P2	1.0	asp	6.936	2.04	15
P2	1.5	asp	10.136	3.48	15
P2	2.0	asp	7.716	2.30	14
P2	24.0	asp	5.481	1.70	15
P2	3.0	asp	5.039	1.43	15
P2	6.0	asp	4.664	1.33	14
P3	0.0	asp	6.212	2.89	14
P3	0.5	asp	5.854	0.97	13
P3	1.0	asp	6.389	1.19	13
P3	1.5	asp	8.299	2.45	14
P3	2.0	asp	8.001	1.64	12
P3	24.0	asp	5.405	1.61	14
P3	3.0	asp	5.719	1.45	13
P3	6.0	asp	4.253	0.92	12
P4	0.0	asp	6.015	1.66	15
P4	0.5	asp	6.559	1.45	14
P4	1.0	asp	7.563	1.69	15
P4	1.5	asp	10.565	2.84	15
P4	2.0	asp	7.849	1.55	14
P4	24.0	asp	5.763	1.02	15
P4	3.0	asp	6.553	3.06	14
P4	6.0	asp	4.731	0.90	14
P5	0.0	asp	5.314	1.26	15
P5	0.5	asp	6.282	1.37	15
P5	1.0	asp	6.295	0.96	15
P5	1.5	asp	12.082	7.02	14

protocol	time	analyte	mean	sd	n_obs
P5	2.0	asp	8.126	1.64	14
P5	24.0	asp	5.179	1.05	15
P5	3.0	asp	4.989	1.36	15
P5	6.0	asp	4.428	1.03	15
P1	0.0	BICARB	25.067	1.53	15
P1	0.5	BICARB	27.133	2.72	15
P1	1.0	BICARB	27.267	3.04	15
P1	1.5	BICARB	26.214	2.01	14
P1	2.0	BICARB	28.667	4.48	15
P1	24.0	BICARB	26.267	1.62	15
P1	3.0	BICARB	27.200	2.34	15
P1	6.0	BICARB	26.333	2.94	15
P2	0.0	BICARB	25.467	1.77	15
P2	0.5	BICARB	18.357	1.74	14
P2	1.0	BICARB	19.400	2.64	15
P2	1.5	BICARB	22.933	2.31	15
P2	2.0	BICARB	24.571	2.71	14
P2	24.0	BICARB	25.200	1.61	15
P2	3.0	BICARB	24.800	1.66	15
P2	6.0	BICARB	24.214	1.97	14
P3	0.0	BICARB	25.357	1.22	14
P3	0.5	BICARB	17.385	2.10	13
P3	1.0	BICARB	19.857	3.57	14
P3	1.5	BICARB	24.357	2.62	14
P3	2.0	BICARB	24.500	3.78	12
P3	24.0	BICARB	25.500	1.45	14
P3	3.0	BICARB	26.000	4.37	14
P3	6.0	BICARB	24.818	1.78	11
P4	0.0	BICARB	25.467	1.36	15
P4	0.5	BICARB	23.929	1.94	14
P4	1.0	BICARB	23.600	2.10	15
P4	1.5	BICARB	23.800	2.57	15
P4	2.0	BICARB	24.929	1.68	14
P4	24.0	BICARB	26.438	1.63	16

protocol	time	analyte	mean	sd	n_obs
P4	3.0	BICARB	25.400	1.68	15
P4	6.0	BICARB	26.000	1.71	14
P5	0.0	BICARB	25.067	2.19	15
P5	0.5	BICARB	17.467	3.34	15
P5	1.0	BICARB	18.067	3.83	15
P5	1.5	BICARB	22.786	2.75	14
P5	2.0	BICARB	23.857	2.41	14
P5	24.0	BICARB	25.643	2.13	14
P5	3.0	BICARB	24.667	2.32	15
P5	6.0	BICARB	24.733	2.12	15
P1	0.0	CHLORIDE	100.067	3.17	15
P1	0.5	CHLORIDE	100.267	2.79	15
P1	1.0	CHLORIDE	100.133	3.23	15
P1	1.5	CHLORIDE	99.571	3.06	14
P1	2.0	CHLORIDE	100.267	3.04	15
P1	24.0	CHLORIDE	99.467	2.17	15
P1	3.0	CHLORIDE	100.067	2.68	15
P1	6.0	CHLORIDE	98.800	2.54	15
P2	0.0	CHLORIDE	99.733	1.58	15
P2	0.5	CHLORIDE	99.538	1.98	13
P2	1.0	CHLORIDE	99.067	2.31	15
P2	1.5	CHLORIDE	98.133	1.88	15
P2	2.0	CHLORIDE	98.571	2.28	14
P2	24.0	CHLORIDE	100.200	1.82	15
P2	3.0	CHLORIDE	96.933	1.83	15
P2	6.0	CHLORIDE	96.571	2.03	14
P3	0.0	CHLORIDE	100.643	2.68	14
P3	0.5	CHLORIDE	100.692	2.14	13
P3	1.0	CHLORIDE	100.643	2.37	14
P3	1.5	CHLORIDE	101.500	2.25	14
P3	2.0	CHLORIDE	101.750	2.42	12
P3	24.0	CHLORIDE	99.643	2.44	14
P3	3.0	CHLORIDE	100.286	1.82	14
P3	6.0	CHLORIDE	97.091	2.26	11

protocol	time	analyte	mean	sd	n_obs
P4	0.0	CHLORIDE	99.600	1.76	15
P4	0.5	CHLORIDE	101.429	2.31	14
P4	1.0	CHLORIDE	99.867	2.07	15
P4	1.5	CHLORIDE	99.000	2.56	15
P4	2.0	CHLORIDE	98.643	2.06	14
P4	24.0	CHLORIDE	100.125	2.90	16
P4	3.0	CHLORIDE	98.333	2.06	15
P4	6.0	CHLORIDE	97.286	2.34	14
P5	0.0	CHLORIDE	100.467	2.36	15
P5	0.5	CHLORIDE	100.467	2.07	15
P5	1.0	CHLORIDE	99.733	1.98	15
P5	1.5	CHLORIDE	98.286	2.09	14
P5	2.0	CHLORIDE	98.071	2.06	14
P5	24.0	CHLORIDE	101.143	2.51	14
P5	3.0	CHLORIDE	98.000	2.20	15
P5	6.0	CHLORIDE	97.400	2.47	15
P1	0.0	citrul	32.917	6.48	15
P1	0.5	citrul	30.921	5.17	15
P1	1.0	citrul	28.970	5.82	15
P1	1.5	citrul	32.128	6.17	15
P1	2.0	citrul	41.649	7.34	14
P1	24.0	citrul	35.491	6.03	15
P1	3.0	citrul	34.669	7.45	15
P1	6.0	citrul	31.099	5.74	15
P2	0.0	citrul	34.735	5.48	15
P2	0.5	citrul	37.791	5.72	14
P2	1.0	citrul	42.616	6.38	15
P2	1.5	citrul	43.933	7.34	15
P2	2.0	citrul	46.157	9.35	14
P2	24.0	citrul	37.303	6.49	15
P2	3.0	citrul	35.389	6.91	15
P2	6.0	citrul	31.222	5.17	14
P3	0.0	citrul	36.100	7.41	14
P3	0.5	citrul	34.555	8.05	13

protocol	time	analyte	mean	sd	n_obs
P3	1.0	citrul	33.455	8.31	13
P3	1.5	citrul	36.759	8.35	14
P3	2.0	citrul	38.453	8.34	12
P3	24.0	citrul	37.434	6.93	14
P3	3.0	citrul	35.455	8.21	13
P3	6.0	citrul	32.857	6.66	12
P4	0.0	citrul	34.881	5.62	15
P4	0.5	citrul	39.144	7.65	14
P4	1.0	citrul	43.833	10.18	15
P4	1.5	citrul	44.041	9.62	15
P4	2.0	citrul	50.536	9.80	14
P4	24.0	citrul	36.596	7.04	15
P4	3.0	citrul	39.016	7.69	14
P4	6.0	citrul	32.331	5.22	14
P5	0.0	citrul	35.125	5.97	15
P5	0.5	citrul	37.366	6.90	15
P5	1.0	citrul	40.739	5.92	15
P5	1.5	citrul	41.354	6.44	14
P5	2.0	citrul	46.314	8.15	14
P5	24.0	citrul	37.275	6.42	15
P5	3.0	citrul	34.209	7.48	15
P5	6.0	citrul	30.746	5.12	15
P1	0.0	CORT	0.548	0.12	15
P1	0.5	CORT	0.462	0.12	15
P1	1.0	CORT	0.422	0.11	14
P1	1.5	CORT	0.346	0.08	14
P1	2.0	CORT	0.312	0.07	15
P1	24.0	CORT	0.480	0.10	15
P1	3.0	CORT	0.275	0.06	15
P1	6.0	CORT	0.283	0.08	15
P2	0.0	CORT	0.450	0.09	15
P2	0.5	CORT	0.536	0.11	14
P2	1.0	CORT	0.663	0.14	15
P2	1.5	CORT	0.638	0.17	15

protocol	time	analyte	mean	sd	n_obs
P2	2.0	CORT	0.558	0.15	14
P2	24.0	CORT	0.414	0.08	15
P2	3.0	CORT	0.419	0.11	15
P2	6.0	CORT	0.184	0.07	14
P3	0.0	CORT	0.515	0.09	14
P3	0.5	CORT	0.554	0.09	13
P3	1.0	CORT	0.725	0.08	14
P3	1.5	CORT	0.687	0.10	14
P3	2.0	CORT	0.569	0.07	12
P3	24.0	CORT	0.436	0.08	14
P3	3.0	CORT	0.463	0.11	14
P3	6.0	CORT	0.180	0.07	11
P4	0.0	CORT	0.483	0.08	15
P4	0.5	CORT	0.433	0.10	14
P4	1.0	CORT	0.382	0.11	15
P4	1.5	CORT	0.308	0.08	15
P4	2.0	CORT	0.282	0.08	14
P4	24.0	CORT	0.460	0.07	15
P4	3.0	CORT	0.252	0.07	15
P4	6.0	CORT	0.253	0.11	14
P5	0.0	CORT	0.500	0.10	15
P5	0.5	CORT	0.509	0.10	15
P5	1.0	CORT	0.645	0.15	15
P5	1.5	CORT	0.584	0.13	14
P5	2.0	CORT	0.484	0.13	14
P5	24.0	CORT	0.437	0.08	14
P5	3.0	CORT	0.369	0.12	15
P5	6.0	CORT	0.249	0.12	15
P1	0.0	ERY	4.893	0.32	15
P1	0.5	ERY	4.907	0.34	14
P1	1.0	ERY	4.867	0.33	15
P1	1.5	ERY	4.907	0.32	15
P1	2.0	ERY	4.880	0.35	15
P1	24.0	ERY	4.920	0.33	15

protocol	time	analyte	mean	sd	n_obs
P1	3.0	ERY	4.847	0.33	15
P1	6.0	ERY	4.847	0.31	15
P2	0.0	ERY	4.750	0.35	14
P2	0.5	ERY	5.092	0.34	13
P2	1.0	ERY	5.050	0.39	14
P2	1.5	ERY	4.757	0.38	14
P2	2.0	ERY	4.662	0.37	13
P2	24.0	ERY	4.620	0.30	15
P2	3.0	ERY	4.714	0.34	14
P2	6.0	ERY	4.708	0.36	13
P3	0.0	ERY	4.685	0.21	13
P3	0.5	ERY	5.092	0.22	12
P3	1.0	ERY	4.954	0.20	13
P3	1.5	ERY	4.769	0.21	13
P3	2.0	ERY	4.667	0.24	12
P3	24.0	ERY	4.529	0.20	14
P3	3.0	ERY	4.662	0.20	13
P3	6.0	ERY	4.570	0.28	10
P4	0.0	ERY	4.657	0.26	14
P4	0.5	ERY	4.977	0.28	13
P4	1.0	ERY	4.893	0.29	14
P4	1.5	ERY	4.650	0.24	14
P4	2.0	ERY	4.623	0.25	13
P4	24.0	ERY	4.719	0.30	16
P4	3.0	ERY	4.650	0.26	14
P4	6.0	ERY	4.608	0.29	13
P5	0.0	ERY	4.687	0.30	15
P5	0.5	ERY	5.173	0.38	15
P5	1.0	ERY	5.047	0.36	15
P5	1.5	ERY	4.713	0.33	15
P5	2.0	ERY	4.640	0.36	15
P5	24.0	ERY	4.614	0.33	14
P5	3.0	ERY	4.627	0.35	15
P5	6.0	ERY	4.700	0.38	15

protocol	time	analyte	mean	sd	n_obs
P1	0.0	gln	581.327	41.06	15
P1	0.5	gln	607.819	50.26	15
P1	1.0	gln	635.520	51.06	15
P1	1.5	gln	957.102	120.57	15
P1	2.0	gln	899.379	133.35	14
P1	24.0	gln	587.312	59.46	15
P1	3.0	gln	624.139	87.38	15
P1	6.0	gln	584.750	48.82	15
P2	0.0	gln	564.054	48.92	15
P2	0.5	gln	575.913	40.10	14
P2	1.0	gln	604.669	40.99	15
P2	1.5	gln	885.463	125.02	15
P2	2.0	gln	799.978	119.71	14
P2	24.0	gln	568.093	43.44	15
P2	3.0	gln	569.228	79.89	15
P2	6.0	gln	542.419	71.05	14
P3	0.0	gln	577.851	41.61	14
P3	0.5	gln	608.334	52.13	13
P3	1.0	gln	618.808	66.79	13
P3	1.5	gln	732.213	91.33	14
P3	2.0	gln	873.640	124.87	12
P3	24.0	gln	563.329	49.35	14
P3	3.0	gln	627.805	80.64	13
P3	6.0	gln	558.720	55.79	12
P4	0.0	gln	553.805	44.72	15
P4	0.5	gln	561.657	63.07	14
P4	1.0	gln	559.068	50.88	15
P4	1.5	gln	971.819	126.28	15
P4	2.0	gln	783.109	103.14	14
P4	24.0	gln	553.690	47.43	15
P4	3.0	gln	575.264	62.14	14
P4	6.0	gln	546.764	43.18	14
P5	0.0	gln	561.694	43.22	15
P5	0.5	gln	564.484	61.74	15

protocol	time	analyte	mean	sd	n_obs
P5	1.0	gln	594.787	75.85	15
P5	1.5	gln	914.552	119.93	14
P5	2.0	gln	848.400	81.51	14
P5	24.0	gln	573.009	33.05	15
P5	3.0	gln	561.343	67.86	15
P5	6.0	gln	552.849	43.08	15
P1	0.0	glu	97.305	36.12	15
P1	0.5	glu	95.527	31.84	15
P1	1.0	glu	80.728	29.52	15
P1	1.5	glu	126.321	36.70	15
P1	2.0	glu	107.578	24.50	14
P1	24.0	glu	104.043	50.39	15
P1	3.0	glu	91.206	22.55	15
P1	6.0	glu	90.983	32.40	15
P2	0.0	glu	114.600	27.50	15
P2	0.5	glu	152.009	39.57	14
P2	1.0	glu	162.585	49.18	15
P2	1.5	glu	190.165	54.80	15
P2	2.0	glu	159.269	50.05	14
P2	24.0	glu	112.403	35.13	15
P2	3.0	glu	105.056	33.45	15
P2	6.0	glu	118.071	41.98	14
P3	0.0	glu	101.094	23.02	14
P3	0.5	glu	128.048	42.45	13
P3	1.0	glu	144.425	36.28	13
P3	1.5	glu	158.046	35.12	14
P3	2.0	glu	151.291	35.37	12
P3	24.0	glu	101.384	22.80	14
P3	3.0	glu	111.579	24.49	13
P3	6.0	glu	101.293	34.44	12
P4	0.0	glu	117.364	25.28	15
P4	0.5	glu	120.456	26.59	14
P4	1.0	glu	136.082	33.23	15
P4	1.5	glu	183.801	38.73	15

protocol	time	analyte	mean	sd	n_obs
P4	2.0	glu	148.316	16.94	14
P4	24.0	glu	115.703	25.76	15
P4	3.0	glu	108.353	28.84	14
P4	6.0	glu	105.541	22.72	14
P5	0.0	glu	105.805	19.14	15
P5	0.5	glu	148.115	46.44	15
P5	1.0	glu	142.530	39.29	15
P5	1.5	glu	192.751	44.27	14
P5	2.0	glu	149.515	23.29	14
P5	24.0	glu	101.995	14.65	15
P5	3.0	glu	102.535	32.57	15
P5	6.0	glu	107.033	32.41	15
P1	0.0	GLU_NS	5.167	0.34	15
P1	1.0	GLU_NS	4.387	0.58	15
P1	2.0	GLU_NS	4.613	0.37	15
P1	6.0	GLU_NS	4.873	0.29	15
P2	0.0	GLU_NS	5.067	0.24	15
P2	1.0	GLU_NS	5.480	0.82	15
P2	2.0	GLU_NS	4.643	0.37	14
P2	6.0	GLU_NS	4.714	0.25	14
P3	0.0	GLU_NS	5.064	0.34	14
P3	1.0	GLU_NS	5.643	1.07	14
P3	2.0	GLU_NS	5.042	0.43	12
P3	6.0	GLU_NS	4.791	0.30	11
P4	0.0	GLU_NS	4.993	0.30	15
P4	1.0	GLU_NS	4.853	0.35	15
P4	2.0	GLU_NS	4.871	0.20	14
P4	6.0	GLU_NS	4.750	0.21	14
P5	0.0	GLU_NS	4.980	0.31	15
P5	1.0	GLU_NS	5.293	1.14	15
P5	2.0	GLU_NS	4.453	0.38	15
P5	6.0	GLU_NS	4.593	0.34	15
P1	0.0	gly	271.173	125.74	15
P1	0.5	gly	269.307	69.58	15

protocol	time	analyte	mean	sd	n_obs
P1	1.0	gly	265.551	51.94	15
P1	1.5	gly	294.417	51.11	15
P1	2.0	gly	261.634	47.17	14
P1	24.0	gly	258.019	80.62	15
P1	3.0	gly	218.632	38.36	15
P1	6.0	gly	213.843	29.18	15
P2	0.0	gly	250.699	46.30	15
P2	0.5	gly	263.844	47.10	14
P2	1.0	gly	272.938	47.58	15
P2	1.5	gly	280.343	65.21	15
P2	2.0	gly	260.611	71.24	14
P2	24.0	gly	227.395	30.47	15
P2	3.0	gly	197.243	40.22	15
P2	6.0	gly	198.733	37.63	14
P3	0.0	gly	245.292	27.73	14
P3	0.5	gly	255.975	29.71	13
P3	1.0	gly	260.328	31.88	13
P3	1.5	gly	246.046	29.98	14
P3	2.0	gly	249.961	24.53	12
P3	24.0	gly	233.429	25.45	14
P3	3.0	gly	201.654	29.63	13
P3	6.0	gly	194.250	24.68	12
P4	0.0	gly	247.385	23.37	15
P4	0.5	gly	271.816	29.98	14
P4	1.0	gly	277.421	31.70	15
P4	1.5	gly	282.663	39.75	15
P4	2.0	gly	244.104	39.27	14
P4	24.0	gly	230.345	32.48	15
P4	3.0	gly	210.064	27.25	14
P4	6.0	gly	203.537	28.42	14
P5	0.0	gly	244.253	36.33	15
P5	0.5	gly	258.062	37.16	15
P5	1.0	gly	262.043	37.23	15
P5	1.5	gly	274.325	49.69	14

protocol	time	analyte	mean	sd	n_obs
P5	2.0	gly	267.431	43.53	14
P5	24.0	gly	216.579	32.92	15
P5	3.0	gly	191.897	34.64	15
P5	6.0	gly	189.237	36.58	15
P1	0.0	HB	9.227	0.48	15
P1	0.5	HB	9.236	0.54	14
P1	1.0	HB	9.187	0.57	15
P1	1.5	HB	9.220	0.58	15
P1	2.0	HB	9.207	0.61	15
P1	24.0	HB	9.233	0.56	15
P1	3.0	HB	9.140	0.56	15
P1	6.0	HB	9.147	0.50	15
P2	0.0	HB	8.836	0.55	14
P2	0.5	HB	9.615	0.52	13
P2	1.0	HB	9.493	0.54	14
P2	1.5	HB	8.950	0.53	14
P2	2.0	HB	8.769	0.53	13
P2	24.0	HB	8.733	0.45	15
P2	3.0	HB	8.807	0.51	14
P2	6.0	HB	8.823	0.54	13
P3	0.0	HB	8.831	0.41	13
P3	0.5	HB	9.600	0.44	12
P3	1.0	HB	9.423	0.47	13
P3	1.5	HB	9.031	0.44	13
P3	2.0	HB	8.750	0.44	12
P3	24.0	HB	8.564	0.40	14
P3	3.0	HB	8.815	0.39	13
P3	6.0	HB	8.670	0.38	10
P4	0.0	HB	8.779	0.50	14
P4	0.5	HB	9.392	0.54	13
P4	1.0	HB	9.279	0.56	14
P4	1.5	HB	8.807	0.44	14
P4	2.0	HB	8.739	0.47	13
P4	24.0	HB	8.853	0.49	15

protocol	time	analyte	mean	sd	n_obs
P4	3.0	HB	8.793	0.52	14
P4	6.0	HB	8.731	0.58	13
P5	0.0	HB	8.773	0.57	15
P5	0.5	HB	9.660	0.50	15
P5	1.0	HB	9.453	0.51	15
P5	1.5	HB	8.853	0.48	15
P5	2.0	HB	8.713	0.51	15
P5	24.0	HB	8.671	0.55	14
P5	3.0	HB	8.707	0.53	15
P5	6.0	HB	8.780	0.57	15
P1	0.0	HT	0.416	0.02	15
P1	0.5	HT	0.415	0.02	14
P1	1.0	HT	0.417	0.02	15
P1	1.5	HT	0.419	0.03	15
P1	2.0	HT	0.416	0.03	15
P1	24.0	HT	0.419	0.02	15
P1	3.0	HT	0.413	0.03	15
P1	6.0	HT	0.411	0.02	15
P2	0.0	HT	0.402	0.02	14
P2	0.5	HT	0.438	0.02	13
P2	1.0	HT	0.432	0.02	14
P2	1.5	HT	0.403	0.02	14
P2	2.0	HT	0.395	0.03	13
P2	24.0	HT	0.399	0.02	15
P2	3.0	HT	0.398	0.02	14
P2	6.0	HT	0.398	0.03	13
P3	0.0	HT	0.405	0.02	13
P3	0.5	HT	0.441	0.02	12
P3	1.0	HT	0.429	0.02	13
P3	1.5	HT	0.410	0.02	13
P3	2.0	HT	0.401	0.02	12
P3	24.0	HT	0.390	0.02	14
P3	3.0	HT	0.400	0.02	13
P3	6.0	HT	0.389	0.02	10

protocol	time	analyte	mean	sd	n_obs
P4	0.0	HT	0.401	0.02	14
P4	0.5	HT	0.425	0.02	13
P4	1.0	HT	0.420	0.02	14
P4	1.5	HT	0.399	0.02	14
P4	2.0	HT	0.398	0.02	13
P4	24.0	HT	0.402	0.02	16
P4	3.0	HT	0.400	0.02	14
P4	6.0	HT	0.395	0.02	13
P5	0.0	HT	0.400	0.02	15
P5	0.5	HT	0.445	0.03	15
P5	1.0	HT	0.436	0.02	15
P5	1.5	HT	0.402	0.02	15
P5	2.0	HT	0.395	0.02	15
P5	24.0	HT	0.399	0.02	14
P5	3.0	HT	0.393	0.02	15
P5	6.0	HT	0.398	0.03	15
P1	0.0	ifabp	841.374	448.27	15
P1	0.5	ifabp	855.446	440.44	15
P1	1.0	ifabp	642.700	344.38	15
P1	1.5	ifabp	435.986	269.16	14
P1	2.0	ifabp	415.095	306.21	15
P1	24.0	ifabp	830.411	455.76	15
P1	3.0	ifabp	679.362	427.39	15
P1	6.0	ifabp	643.611	400.08	15
P2	0.0	ifabp	742.860	341.13	15
P2	0.5	ifabp	910.381	270.55	14
P2	1.0	ifabp	1262.841	512.74	14
P2	1.5	ifabp	1098.044	585.32	15
P2	2.0	ifabp	788.346	448.48	14
P2	24.0	ifabp	816.947	381.08	14
P2	3.0	ifabp	468.897	292.74	15
P2	6.0	ifabp	505.601	250.98	14
P3	0.0	ifabp	689.776	309.97	14
P3	0.5	ifabp	917.689	349.19	14

protocol	time	analyte	mean	sd	n_obs
P3	1.0	ifabp	1559.869	658.93	14
P3	1.5	ifabp	1443.720	901.23	13
P3	2.0	ifabp	956.983	699.57	12
P3	24.0	ifabp	900.535	563.24	14
P3	3.0	ifabp	448.870	252.01	14
P3	6.0	ifabp	537.664	328.94	12
P4	0.0	ifabp	698.842	369.10	15
P4	0.5	ifabp	700.636	377.15	13
P4	1.0	ifabp	598.556	360.26	15
P4	1.5	ifabp	387.107	204.16	15
P4	2.0	ifabp	346.737	164.66	14
P4	24.0	ifabp	646.478	367.54	10
P4	3.0	ifabp	505.551	300.57	15
P4	6.0	ifabp	583.784	275.07	14
P5	0.0	ifabp	720.480	359.21	15
P5	0.5	ifabp	901.460	402.89	14
P5	1.0	ifabp	1198.754	714.66	15
P5	1.5	ifabp	807.915	513.93	14
P5	2.0	ifabp	535.861	306.07	14
P5	24.0	ifabp	745.058	360.87	14
P5	3.0	ifabp	424.613	243.32	15
P5	6.0	ifabp	511.252	324.59	15
P1	0.0	ile	64.292	8.93	15
P1	0.5	ile	73.086	12.16	15
P1	1.0	ile	70.355	9.45	15
P1	1.5	ile	62.755	8.41	15
P1	2.0	ile	54.145	9.67	14
P1	24.0	ile	75.578	9.19	15
P1	3.0	ile	53.579	7.63	15
P1	6.0	ile	64.315	5.79	15
P2	0.0	ile	70.783	9.11	15
P2	0.5	ile	79.640	8.16	14
P2	1.0	ile	83.923	12.38	15
P2	1.5	ile	72.600	10.99	15

protocol	time	analyte	mean	sd	n_obs
P2	2.0	ile	62.432	11.13	14
P2	24.0	ile	78.295	12.31	15
P2	3.0	ile	56.784	8.03	15
P2	6.0	ile	77.864	11.53	14
P3	0.0	ile	70.399	11.59	14
P3	0.5	ile	75.305	12.19	13
P3	1.0	ile	75.907	13.72	13
P3	1.5	ile	75.050	11.52	14
P3	2.0	ile	69.097	11.99	12
P3	24.0	ile	76.672	12.17	14
P3	3.0	ile	56.326	8.27	13
P3	6.0	ile	72.263	8.64	12
P4	0.0	ile	67.935	10.84	15
P4	0.5	ile	83.227	13.86	14
P4	1.0	ile	81.524	12.82	15
P4	1.5	ile	66.907	11.81	15
P4	2.0	ile	57.249	12.11	14
P4	24.0	ile	75.625	11.97	15
P4	3.0	ile	53.668	11.03	14
P4	6.0	ile	69.044	8.50	14
P5	0.0	ile	71.243	9.64	15
P5	0.5	ile	79.369	10.23	15
P5	1.0	ile	81.933	10.11	15
P5	1.5	ile	73.488	11.93	14
P5	2.0	ile	63.816	11.13	14
P5	24.0	ile	82.261	12.46	15
P5	3.0	ile	62.049	17.79	15
P5	6.0	ile	83.115	21.79	15
P1	0.0	INSULINE	5.920	1.54	15
P1	0.5	INSULINE	24.460	11.31	15
P1	1.0	INSULINE	10.240	4.37	15
P1	1.5	INSULINE	11.350	5.09	14
P1	2.0	INSULINE	7.213	2.34	15
P1	24.0	INSULINE	6.540	1.64	15

protocol	time	analyte	mean	sd	n_obs
P1	3.0	INSULINE	4.693	1.93	15
P1	6.0	INSULINE	3.460	1.03	15
P2	0.0	INSULINE	5.620	2.23	15
P2	0.5	INSULINE	3.643	1.82	14
P2	1.0	INSULINE	3.420	2.60	15
P2	1.5	INSULINE	13.280	5.75	15
P2	2.0	INSULINE	6.464	2.97	14
P2	24.0	INSULINE	5.133	1.22	15
P2	3.0	INSULINE	4.167	2.45	15
P2	6.0	INSULINE	2.771	1.01	14
P3	0.0	INSULINE	6.036	1.82	14
P3	0.5	INSULINE	3.815	1.97	13
P3	1.0	INSULINE	3.329	2.71	14
P3	1.5	INSULINE	20.293	10.91	14
P3	2.0	INSULINE	13.250	4.21	12
P3	24.0	INSULINE	5.550	1.71	14
P3	3.0	INSULINE	5.779	1.98	14
P3	6.0	INSULINE	3.027	1.11	11
P4	0.0	INSULINE	6.673	2.56	15
P4	0.5	INSULINE	8.500	3.57	14
P4	1.0	INSULINE	3.700	2.07	15
P4	1.5	INSULINE	11.507	5.79	15
P4	2.0	INSULINE	7.050	3.12	14
P4	24.0	INSULINE	5.213	2.12	16
P4	3.0	INSULINE	4.720	1.84	15
P4	6.0	INSULINE	2.986	1.05	14
P5	0.0	INSULINE	6.253	2.68	15
P5	0.5	INSULINE	5.080	2.49	15
P5	1.0	INSULINE	3.800	3.58	15
P5	1.5	INSULINE	12.786	9.81	14
P5	2.0	INSULINE	6.107	4.28	14
P5	24.0	INSULINE	6.300	1.90	14
P5	3.0	INSULINE	3.100	1.90	15
P5	6.0	INSULINE	3.013	1.46	15

protocol	time	analyte	mean	sd	n_obs
P1	0.0	KREAT	81.600	11.49	15
P1	0.5	KREAT	79.600	9.44	15
P1	1.0	KREAT	79.600	10.01	15
P1	1.5	KREAT	77.143	9.44	14
P1	2.0	KREAT	77.533	10.34	15
P1	24.0	KREAT	79.933	10.10	15
P1	3.0	KREAT	75.333	9.74	15
P1	6.0	KREAT	74.267	8.40	15
P2	0.0	KREAT	81.600	9.43	15
P2	0.5	KREAT	93.357	10.94	14
P2	1.0	KREAT	103.533	15.04	15
P2	1.5	KREAT	97.533	11.95	15
P2	2.0	KREAT	94.500	8.33	14
P2	24.0	KREAT	82.800	9.02	15
P2	3.0	KREAT	87.533	10.16	15
P2	6.0	KREAT	81.643	8.72	14
P3	0.0	KREAT	84.214	9.33	14
P3	0.5	KREAT	98.385	12.83	13
P3	1.0	KREAT	107.571	17.93	14
P3	1.5	KREAT	97.357	14.63	14
P3	2.0	KREAT	95.917	13.81	12
P3	24.0	KREAT	82.857	8.15	14
P3	3.0	KREAT	91.714	11.36	14
P3	6.0	KREAT	83.727	12.27	11
P4	0.0	KREAT	83.467	9.65	15
P4	0.5	KREAT	85.643	11.15	14
P4	1.0	KREAT	85.733	12.13	15
P4	1.5	KREAT	83.867	11.88	15
P4	2.0	KREAT	83.714	11.25	14
P4	24.0	KREAT	81.750	8.87	16
P4	3.0	KREAT	81.933	11.44	15
P4	6.0	KREAT	79.000	9.77	14
P5	0.0	KREAT	82.133	9.23	15
P5	0.5	KREAT	91.400	13.03	15

protocol	time	analyte	mean	sd	n_obs
P5	1.0	KREAT	101.667	16.93	15
P5	1.5	KREAT	94.143	13.24	14
P5	2.0	KREAT	91.929	11.92	14
P5	24.0	KREAT	83.286	8.20	14
P5	3.0	KREAT	86.600	10.51	15
P5	6.0	KREAT	80.600	10.34	15
P1	0.0	leu	122.333	12.56	15
P1	0.5	leu	136.405	19.66	15
P1	1.0	leu	130.520	12.34	15
P1	1.5	leu	117.849	10.96	15
P1	2.0	leu	101.465	13.59	14
P1	24.0	leu	130.289	11.50	15
P1	3.0	leu	99.190	10.37	15
P1	6.0	leu	122.911	13.03	15
P2	0.0	leu	125.771	11.39	15
P2	0.5	leu	140.815	12.09	14
P2	1.0	leu	149.446	16.92	15
P2	1.5	leu	131.596	16.55	15
P2	2.0	leu	113.485	17.00	14
P2	24.0	leu	131.262	16.41	15
P2	3.0	leu	100.072	13.24	15
P2	6.0	leu	133.068	15.86	14
P3	0.0	leu	124.434	15.34	14
P3	0.5	leu	133.461	15.13	13
P3	1.0	leu	136.811	18.68	13
P3	1.5	leu	137.367	16.97	14
P3	2.0	leu	124.579	18.55	12
P3	24.0	leu	126.958	14.09	14
P3	3.0	leu	98.988	11.96	13
P3	6.0	leu	122.147	11.56	12
P4	0.0	leu	123.935	15.30	15
P4	0.5	leu	148.069	16.84	14
P4	1.0	leu	144.186	16.87	15
P4	1.5	leu	120.520	15.60	15

protocol	time	analyte	mean	sd	n_obs
P4	2.0	leu	101.729	14.95	14
P4	24.0	leu	129.824	17.15	15
P4	3.0	leu	93.206	13.25	14
P4	6.0	leu	121.796	11.48	14
P5	0.0	leu	127.034	16.42	15
P5	0.5	leu	140.112	17.03	15
P5	1.0	leu	145.542	16.01	15
P5	1.5	leu	132.894	20.80	14
P5	2.0	leu	112.756	20.82	14
P5	24.0	leu	134.416	17.03	15
P5	3.0	leu	106.799	31.70	15
P5	6.0	leu	140.962	41.02	15
P1	0.0	LEUCO	5.253	0.84	15
P1	0.5	LEUCO	5.064	0.80	14
P1	1.0	LEUCO	5.547	1.13	15
P1	1.5	LEUCO	5.787	1.07	15
P1	2.0	LEUCO	6.013	1.19	15
P1	24.0	LEUCO	5.207	0.90	15
P1	3.0	LEUCO	5.993	1.22	15
P1	6.0	LEUCO	6.200	1.14	15
P2	0.0	LEUCO	5.021	1.02	14
P2	0.5	LEUCO	9.323	2.01	13
P2	1.0	LEUCO	9.600	2.21	14
P2	1.5	LEUCO	7.186	2.05	14
P2	2.0	LEUCO	9.000	2.27	13
P2	24.0	LEUCO	4.787	1.14	15
P2	3.0	LEUCO	11.386	2.23	14
P2	6.0	LEUCO	9.331	1.88	13
P3	0.0	LEUCO	4.962	1.02	13
P3	0.5	LEUCO	9.300	1.69	12
P3	1.0	LEUCO	9.185	1.99	13
P3	1.5	LEUCO	7.392	1.89	13
P3	2.0	LEUCO	9.583	2.60	12
P3	24.0	LEUCO	5.007	0.96	14

protocol	time	analyte	mean	sd	n_obs
P3	3.0	LEUCO	11.292	2.44	13
P3	6.0	LEUCO	9.000	1.76	10
P4	0.0	LEUCO	4.836	0.76	14
P4	0.5	LEUCO	7.362	1.02	13
P4	1.0	LEUCO	7.786	1.36	14
P4	1.5	LEUCO	5.536	0.87	14
P4	2.0	LEUCO	6.431	1.12	13
P4	24.0	LEUCO	4.781	1.12	16
P4	3.0	LEUCO	7.521	1.40	14
P4	6.0	LEUCO	6.808	1.34	13
P5	0.0	LEUCO	4.707	0.65	15
P5	0.5	LEUCO	9.420	1.40	15
P5	1.0	LEUCO	9.380	1.48	15
P5	1.5	LEUCO	7.227	3.42	15
P5	2.0	LEUCO	9.873	4.32	15
P5	24.0	LEUCO	4.936	0.91	14
P5	3.0	LEUCO	11.953	3.06	15
P5	6.0	LEUCO	9.807	2.20	15
P1	0.0	LYMFO_ABS_CORR	1.960	0.50	15
P1	0.5	LYMFO_ABS_CORR	1.650	0.44	14
P1	1.0	LYMFO_ABS_CORR	1.653	0.43	15
P1	1.5	LYMFO_ABS_CORR	1.633	0.45	15
P1	2.0	LYMFO_ABS_CORR	1.720	0.46	15
P1	24.0	LYMFO_ABS_CORR	1.847	0.47	15
P1	3.0	LYMFO_ABS_CORR	1.721	0.46	14
P1	6.0	LYMFO_ABS_CORR	2.087	0.56	15
P2	0.0	LYMFO_ABS_CORR	1.993	0.43	14
P2	0.5	LYMFO_ABS_CORR	4.093	1.14	14
P2	1.0	LYMFO_ABS_CORR	4.307	1.24	15
P2	1.5	LYMFO_ABS_CORR	1.473	0.43	15
P2	2.0	LYMFO_ABS_CORR	1.321	0.36	14
P2	24.0	LYMFO_ABS_CORR	1.787	0.51	15
P2	3.0	LYMFO_ABS_CORR	1.350	0.40	14
P2	6.0	LYMFO_ABS_CORR	2.062	0.62	13

protocol	time	analyte	mean	sd	n_obs
P3	0.0	LYMFO_ABS_CORR	1.857	0.63	14
P3	0.5	LYMFO_ABS_CORR	4.046	1.28	13
P3	1.0	LYMFO_ABS_CORR	3.679	1.27	14
P3	1.5	LYMFO_ABS_CORR	1.393	0.39	14
P3	2.0	LYMFO_ABS_CORR	1.200	0.40	13
P3	24.0	LYMFO_ABS_CORR	1.871	0.58	14
P3	3.0	LYMFO_ABS_CORR	1.250	0.42	14
P3	6.0	LYMFO_ABS_CORR	1.818	0.67	11
P4	0.0	LYMFO_ABS_CORR	1.813	0.47	15
P4	0.5	LYMFO_ABS_CORR	2.736	0.74	14
P4	1.0	LYMFO_ABS_CORR	2.720	0.84	15
P4	1.5	LYMFO_ABS_CORR	1.533	0.45	15
P4	2.0	LYMFO_ABS_CORR	1.629	0.54	14
P4	24.0	LYMFO_ABS_CORR	1.819	0.56	16
P4	3.0	LYMFO_ABS_CORR	1.853	0.53	15
P4	6.0	LYMFO_ABS_CORR	2.121	0.66	14
P5	0.0	LYMFO_ABS_CORR	1.860	0.42	15
P5	0.5	LYMFO_ABS_CORR	4.400	1.01	15
P5	1.0	LYMFO_ABS_CORR	4.233	1.19	15
P5	1.5	LYMFO_ABS_CORR	1.507	0.70	15
P5	2.0	LYMFO_ABS_CORR	1.436	0.72	14
P5	24.0	LYMFO_ABS_CORR	1.843	0.50	14
P5	3.0	LYMFO_ABS_CORR	1.367	0.36	15
P5	6.0	LYMFO_ABS_CORR	1.900	0.45	14
P1	0.0	lys	152.277	32.49	15
P1	0.5	lys	161.801	35.48	15
P1	1.0	lys	158.131	27.39	15
P1	1.5	lys	156.690	30.46	15
P1	2.0	lys	150.622	28.42	14
P1	24.0	lys	162.783	26.27	15
P1	3.0	lys	136.151	22.04	15
P1	6.0	lys	140.955	23.97	15
P2	0.0	lys	156.515	29.70	15
P2	0.5	lys	170.249	30.00	14

protocol	time	analyte	mean	sd	n_obs
P2	1.0	lys	173.588	30.16	15
P2	1.5	lys	154.339	26.32	15
P2	2.0	lys	150.647	28.95	14
P2	24.0	lys	154.519	22.76	15
P2	3.0	lys	125.530	23.05	15
P2	6.0	lys	136.037	18.75	14
P3	0.0	lys	154.567	30.42	14
P3	0.5	lys	163.730	28.86	13
P3	1.0	lys	165.697	35.42	13
P3	1.5	lys	153.881	28.89	14
P3	2.0	lys	144.929	32.23	12
P3	24.0	lys	150.247	26.14	14
P3	3.0	lys	124.646	20.92	13
P3	6.0	lys	125.573	20.35	12
P4	0.0	lys	155.045	28.92	15
P4	0.5	lys	170.804	28.05	14
P4	1.0	lys	167.423	28.98	15
P4	1.5	lys	154.503	27.38	15
P4	2.0	lys	147.427	26.67	14
P4	24.0	lys	157.897	26.00	15
P4	3.0	lys	129.151	21.18	14
P4	6.0	lys	137.047	15.54	14
P5	0.0	lys	153.394	26.59	15
P5	0.5	lys	163.511	27.67	15
P5	1.0	lys	167.381	26.75	15
P5	1.5	lys	149.930	25.54	14
P5	2.0	lys	147.046	29.00	14
P5	24.0	lys	154.286	24.32	15
P5	3.0	lys	122.217	19.99	15
P5	6.0	lys	131.184	19.18	15
P1	0.0	meth	23.773	6.99	15
P1	0.5	meth	26.807	3.91	15
P1	1.0	meth	26.063	3.15	15
P1	1.5	meth	25.107	3.41	15

protocol	time	analyte	mean	sd	n_obs
P1	2.0	meth	22.431	3.08	14
P1	24.0	meth	29.890	5.88	15
P1	3.0	meth	20.362	3.14	15
P1	6.0	meth	21.497	3.21	15
P2	0.0	meth	27.869	2.77	15
P2	0.5	meth	31.156	4.40	14
P2	1.0	meth	34.325	4.20	15
P2	1.5	meth	31.870	3.72	15
P2	2.0	meth	27.918	3.75	14
P2	24.0	meth	28.413	2.30	15
P2	3.0	meth	21.372	2.99	15
P2	6.0	meth	21.678	2.62	14
P3	0.0	meth	26.659	3.29	14
P3	0.5	meth	29.364	3.82	13
P3	1.0	meth	32.672	4.64	13
P3	1.5	meth	30.766	3.81	14
P3	2.0	meth	27.744	3.77	12
P3	24.0	meth	30.069	2.55	14
P3	3.0	meth	20.729	3.52	13
P3	6.0	meth	20.293	2.88	12
P4	0.0	meth	26.263	2.87	15
P4	0.5	meth	29.181	3.53	14
P4	1.0	meth	29.733	3.06	15
P4	1.5	meth	27.303	3.52	15
P4	2.0	meth	24.151	3.52	14
P4	24.0	meth	28.274	3.52	15
P4	3.0	meth	21.063	2.77	14
P4	6.0	meth	21.901	2.58	14
P5	0.0	meth	27.229	2.28	15
P5	0.5	meth	30.228	2.59	15
P5	1.0	meth	32.909	3.28	15
P5	1.5	meth	31.326	3.11	14
P5	2.0	meth	28.148	3.06	14
P5	24.0	meth	28.304	3.14	15

protocol	time	analyte	mean	sd	n_obs
P5	3.0	meth	20.961	2.33	15
P5	6.0	meth	20.525	3.30	15
P1	0.0	MONO_ABS	0.360	0.09	15
P1	0.5	MONO_ABS	0.350	0.08	14
P1	1.0	MONO_ABS	0.393	0.08	15
P1	1.5	MONO_ABS	0.380	0.07	15
P1	2.0	MONO_ABS	0.413	0.07	15
P1	24.0	MONO_ABS	0.367	0.09	15
P1	3.0	MONO_ABS	0.360	0.08	15
P1	6.0	MONO_ABS	0.347	0.07	15
P2	0.0	MONO_ABS	0.347	0.10	15
P2	0.5	MONO_ABS	0.679	0.20	14
P2	1.0	MONO_ABS	0.653	0.15	15
P2	1.5	MONO_ABS	0.407	0.15	15
P2	2.0	MONO_ABS	0.507	0.18	14
P2	24.0	MONO_ABS	0.347	0.11	15
P2	3.0	MONO_ABS	0.547	0.19	15
P2	6.0	MONO_ABS	0.393	0.12	14
P3	0.0	MONO_ABS	0.357	0.08	14
P3	0.5	MONO_ABS	0.646	0.14	13
P3	1.0	MONO_ABS	0.579	0.15	14
P3	1.5	MONO_ABS	0.329	0.11	14
P3	2.0	MONO_ABS	0.477	0.17	13
P3	24.0	MONO_ABS	0.357	0.10	14
P3	3.0	MONO_ABS	0.521	0.15	14
P3	6.0	MONO_ABS	0.400	0.10	11
P4	0.0	MONO_ABS	0.360	0.12	15
P4	0.5	MONO_ABS	0.564	0.22	14
P4	1.0	MONO_ABS	0.593	0.20	15
P4	1.5	MONO_ABS	0.360	0.13	15
P4	2.0	MONO_ABS	0.407	0.08	14
P4	24.0	MONO_ABS	0.350	0.08	16
P4	3.0	MONO_ABS	0.433	0.13	15
P4	6.0	MONO_ABS	0.350	0.06	14

protocol	time	analyte	mean	sd	n_obs
P5	0.0	MONO_ABS	0.333	0.08	15
P5	0.5	MONO_ABS	0.673	0.13	15
P5	1.0	MONO_ABS	0.620	0.11	15
P5	1.5	MONO_ABS	0.380	0.12	15
P5	2.0	MONO_ABS	0.487	0.17	15
P5	24.0	MONO_ABS	0.343	0.08	14
P5	3.0	MONO_ABS	0.533	0.14	15
P5	6.0	MONO_ABS	0.400	0.12	15
P1	0.0	NEUTRO_ABS	2.693	0.70	15
P1	0.5	NEUTRO_ABS	2.886	0.72	14
P1	1.0	NEUTRO_ABS	3.320	1.08	15
P1	1.5	NEUTRO_ABS	3.567	1.15	15
P1	2.0	NEUTRO_ABS	3.693	1.31	15
P1	24.0	NEUTRO_ABS	2.753	0.74	15
P1	3.0	NEUTRO_ABS	3.693	1.25	15
P1	6.0	NEUTRO_ABS	3.547	1.11	15
P2	0.0	NEUTRO_ABS	2.627	0.68	15
P2	0.5	NEUTRO_ABS	4.350	1.14	14
P2	1.0	NEUTRO_ABS	4.387	1.21	15
P2	1.5	NEUTRO_ABS	5.333	1.80	15
P2	2.0	NEUTRO_ABS	7.593	2.81	14
P2	24.0	NEUTRO_ABS	2.453	0.75	15
P2	3.0	NEUTRO_ABS	9.853	2.79	15
P2	6.0	NEUTRO_ABS	7.129	2.05	14
P3	0.0	NEUTRO_ABS	2.550	0.46	14
P3	0.5	NEUTRO_ABS	4.308	0.83	13
P3	1.0	NEUTRO_ABS	4.614	1.19	14
P3	1.5	NEUTRO_ABS	5.357	1.94	14
P3	2.0	NEUTRO_ABS	7.908	2.54	13
P3	24.0	NEUTRO_ABS	2.600	0.56	14
P3	3.0	NEUTRO_ABS	9.671	2.17	14
P3	6.0	NEUTRO_ABS	6.755	1.15	11
P4	0.0	NEUTRO_ABS	2.447	0.46	15
P4	0.5	NEUTRO_ABS	3.764	0.62	14

protocol	time	analyte	mean	sd	n_obs
P4	1.0	NEUTRO_ABS	4.180	0.77	15
P4	1.5	NEUTRO_ABS	3.480	0.72	15
P4	2.0	NEUTRO_ABS	4.279	1.05	14
P4	24.0	NEUTRO_ABS	2.394	0.71	16
P4	3.0	NEUTRO_ABS	5.167	1.16	15
P4	6.0	NEUTRO_ABS	4.250	0.88	14
P5	0.0	NEUTRO_ABS	2.313	0.44	15
P5	0.5	NEUTRO_ABS	4.033	0.75	15
P5	1.0	NEUTRO_ABS	4.233	0.88	15
P5	1.5	NEUTRO_ABS	5.187	3.33	15
P5	2.0	NEUTRO_ABS	7.840	3.86	15
P5	24.0	NEUTRO_ABS	2.579	0.67	14
P5	3.0	NEUTRO_ABS	9.953	2.99	15
P5	6.0	NEUTRO_ABS	7.380	2.19	15
P1	0.0	pheala	54.634	6.27	15
P1	0.5	pheala	61.791	9.59	15
P1	1.0	pheala	61.563	5.89	15
P1	1.5	pheala	54.876	6.16	15
P1	2.0	pheala	47.805	6.99	14
P1	24.0	pheala	56.921	7.62	15
P1	3.0	pheala	45.890	5.34	15
P1	6.0	pheala	51.043	6.45	15
P2	0.0	pheala	56.884	7.51	15
P2	0.5	pheala	65.539	7.51	14
P2	1.0	pheala	74.670	10.13	15
P2	1.5	pheala	64.827	8.49	15
P2	2.0	pheala	57.262	8.98	14
P2	24.0	pheala	55.936	7.72	15
P2	3.0	pheala	48.739	7.48	15
P2	6.0	pheala	53.924	6.39	14
P3	0.0	pheala	54.996	6.58	14
P3	0.5	pheala	62.772	6.92	13
P3	1.0	pheala	69.712	9.90	13
P3	1.5	pheala	66.536	7.99	14

protocol	time	analyte	mean	sd	n_obs
P3	2.0	pheala	60.248	8.23	12
P3	24.0	pheala	57.286	7.66	14
P3	3.0	pheala	50.373	5.73	13
P3	6.0	pheala	53.458	6.55	12
P4	0.0	pheala	55.955	6.68	15
P4	0.5	pheala	66.379	7.35	14
P4	1.0	pheala	67.224	7.54	15
P4	1.5	pheala	55.965	7.43	15
P4	2.0	pheala	48.561	6.21	14
P4	24.0	pheala	56.953	7.97	15
P4	3.0	pheala	44.199	7.09	14
P4	6.0	pheala	50.576	6.52	14
P5	0.0	pheala	55.789	8.36	15
P5	0.5	pheala	63.676	9.20	15
P5	1.0	pheala	70.226	10.35	15
P5	1.5	pheala	63.414	8.61	14
P5	2.0	pheala	53.442	5.17	14
P5	24.0	pheala	56.129	8.37	15
P5	3.0	pheala	46.735	7.22	15
P5	6.0	pheala	51.585	7.32	15
P1	0.0	POTASSIUM	4.253	0.38	15
P1	0.5	POTASSIUM	4.207	0.33	15
P1	1.0	POTASSIUM	4.300	0.28	15
P1	1.5	POTASSIUM	4.393	0.20	14
P1	2.0	POTASSIUM	4.460	0.20	15
P1	24.0	POTASSIUM	4.320	0.32	15
P1	3.0	POTASSIUM	4.380	0.22	15
P1	6.0	POTASSIUM	4.140	0.34	15
P2	0.0	POTASSIUM	4.140	0.28	15
P2	0.5	POTASSIUM	5.271	0.38	14
P2	1.0	POTASSIUM	5.313	0.32	15
P2	1.5	POTASSIUM	4.393	0.22	15
P2	2.0	POTASSIUM	4.679	0.23	14
P2	24.0	POTASSIUM	4.373	0.25	15

protocol	time	analyte	mean	sd	n_obs
P2	3.0	POTASSIUM	4.287	0.30	15
P2	6.0	POTASSIUM	4.093	0.25	14
P3	0.0	POTASSIUM	4.179	0.27	14
P3	0.5	POTASSIUM	5.292	0.31	13
P3	1.0	POTASSIUM	5.129	0.49	14
P3	1.5	POTASSIUM	4.321	0.16	14
P3	2.0	POTASSIUM	4.542	0.22	12
P3	24.0	POTASSIUM	4.264	0.21	14
P3	3.0	POTASSIUM	4.457	0.29	14
P3	6.0	POTASSIUM	4.091	0.23	11
P4	0.0	POTASSIUM	4.140	0.19	15
P4	0.5	POTASSIUM	4.936	0.19	14
P4	1.0	POTASSIUM	4.987	0.25	15
P4	1.5	POTASSIUM	4.393	0.26	15
P4	2.0	POTASSIUM	4.429	0.23	14
P4	24.0	POTASSIUM	4.456	0.26	16
P4	3.0	POTASSIUM	4.373	0.22	15
P4	6.0	POTASSIUM	4.029	0.24	14
P5	0.0	POTASSIUM	4.160	0.32	15
P5	0.5	POTASSIUM	5.193	0.29	15
P5	1.0	POTASSIUM	5.227	0.36	15
P5	1.5	POTASSIUM	4.393	0.32	14
P5	2.0	POTASSIUM	4.650	0.35	14
P5	24.0	POTASSIUM	4.293	0.30	14
P5	3.0	POTASSIUM	4.453	0.29	15
P5	6.0	POTASSIUM	4.133	0.26	15
P2	15.0	RPE	13.067	2.05	15
P2	30.0	RPE	15.929	1.98	15
P2	45.0	RPE	17.200	1.74	15
P2	60.0	RPE	17.633	2.14	15
P3	15.0	RPE	13.214	1.48	15
P3	30.0	RPE	16.643	1.98	15
P3	45.0	RPE	17.643	1.98	15
P3	60.0	RPE	18.500	1.02	15

protocol	time	analyte	mean	sd	n_obs
P4	15.0	RPE	10.400	1.80	15
P4	30.0	RPE	11.267	1.75	15
P4	45.0	RPE	11.600	1.59	15
P4	60.0	RPE	11.667	1.76	15
P5	15.0	RPE	14.267	1.83	15
P5	30.0	RPE	15.667	2.50	15
P5	45.0	RPE	16.333	2.44	15
P5	60.0	RPE	17.214	2.78	14
P1	0.0	ser	110.137	20.69	15
P1	0.5	ser	122.405	26.92	15
P1	1.0	ser	115.221	20.39	15
P1	1.5	ser	164.895	40.34	15
P1	2.0	ser	134.039	29.98	14
P1	24.0	ser	108.899	18.30	15
P1	3.0	ser	97.735	18.24	15
P1	6.0	ser	95.578	15.22	15
P2	0.0	ser	109.785	16.97	15
P2	0.5	ser	113.911	21.46	14
P2	1.0	ser	120.974	22.24	15
P2	1.5	ser	155.337	33.57	15
P2	2.0	ser	131.510	32.18	14
P2	24.0	ser	110.520	15.30	15
P2	3.0	ser	89.275	16.02	15
P2	6.0	ser	90.551	14.52	14
P3	0.0	ser	109.586	21.17	14
P3	0.5	ser	106.121	20.33	13
P3	1.0	ser	112.288	20.53	13
P3	1.5	ser	128.254	21.73	14
P3	2.0	ser	131.744	21.63	12
P3	24.0	ser	109.442	16.08	14
P3	3.0	ser	91.072	20.43	13
P3	6.0	ser	86.987	15.78	12
P4	0.0	ser	107.499	14.70	15
P4	0.5	ser	123.148	17.56	14

protocol	time	analyte	mean	sd	n_obs
P4	1.0	ser	124.685	19.28	15
P4	1.5	ser	163.015	34.77	15
P4	2.0	ser	122.660	23.10	14
P4	24.0	ser	105.415	17.82	15
P4	3.0	ser	94.545	13.81	14
P4	6.0	ser	91.038	13.16	14
P5	0.0	ser	108.352	17.43	15
P5	0.5	ser	112.443	19.50	15
P5	1.0	ser	117.523	21.31	15
P5	1.5	ser	155.390	34.51	14
P5	2.0	ser	137.091	28.69	14
P5	24.0	ser	106.727	17.29	15
P5	3.0	ser	87.053	15.78	15
P5	6.0	ser	88.150	16.86	15
P1	0.0	SODIUM	139.200	4.31	15
P1	0.5	SODIUM	138.867	3.91	15
P1	1.0	SODIUM	138.667	4.17	15
P1	1.5	SODIUM	138.214	3.56	14
P1	2.0	SODIUM	138.067	3.54	15
P1	24.0	SODIUM	138.200	2.48	15
P1	3.0	SODIUM	137.800	3.76	15
P1	6.0	SODIUM	138.333	3.29	15
P2	0.0	SODIUM	138.400	2.56	15
P2	0.5	SODIUM	139.643	2.17	14
P2	1.0	SODIUM	139.333	3.15	15
P2	1.5	SODIUM	136.733	2.19	15
P2	2.0	SODIUM	136.500	2.56	14
P2	24.0	SODIUM	138.933	2.71	15
P2	3.0	SODIUM	135.133	2.50	15
P2	6.0	SODIUM	136.071	2.34	14
P3	0.0	SODIUM	138.571	2.90	14
P3	0.5	SODIUM	140.692	2.50	13
P3	1.0	SODIUM	141.286	2.09	14
P3	1.5	SODIUM	140.429	2.17	14

protocol	time	analyte	mean	sd	n_obs
P3	2.0	SODIUM	139.417	2.47	12
P3	24.0	SODIUM	138.000	2.72	14
P3	3.0	SODIUM	138.571	2.53	14
P3	6.0	SODIUM	135.909	2.43	11
P4	0.0	SODIUM	138.400	2.20	15
P4	0.5	SODIUM	139.786	2.78	14
P4	1.0	SODIUM	137.933	2.94	15
P4	1.5	SODIUM	137.267	3.04	15
P4	2.0	SODIUM	136.429	2.56	14
P4	24.0	SODIUM	139.062	2.82	16
P4	3.0	SODIUM	136.400	2.13	15
P4	6.0	SODIUM	136.643	2.79	14
P5	0.0	SODIUM	139.067	2.34	15
P5	0.5	SODIUM	141.067	1.91	15
P5	1.0	SODIUM	140.867	2.56	15
P5	1.5	SODIUM	137.214	2.49	14
P5	2.0	SODIUM	136.214	1.63	14
P5	24.0	SODIUM	139.214	2.99	14
P5	3.0	SODIUM	136.533	2.33	15
P5	6.0	SODIUM	136.867	2.62	15
P1	0.0	tau	108.993	44.16	15
P1	0.5	tau	100.521	36.98	15
P1	1.0	tau	96.034	36.80	15
P1	1.5	tau	116.768	37.86	15
P1	2.0	tau	106.074	31.90	14
P1	24.0	tau	107.864	49.35	15
P1	3.0	tau	101.555	30.97	15
P1	6.0	tau	101.153	36.37	15
P2	0.0	tau	131.534	32.56	15
P2	0.5	tau	205.882	49.88	14
P2	1.0	tau	218.251	55.94	15
P2	1.5	tau	175.149	45.43	15
P2	2.0	tau	174.017	45.91	14
P2	24.0	tau	119.865	30.87	15

protocol	time	analyte	mean	sd	n_obs
P2	3.0	tau	140.087	41.51	15
P2	6.0	tau	134.881	44.35	14
P3	0.0	tau	114.461	27.01	14
P3	0.5	tau	164.887	50.96	13
P3	1.0	tau	175.897	50.31	13
P3	1.5	tau	143.324	29.98	14
P3	2.0	tau	141.351	31.45	12
P3	24.0	tau	117.095	28.02	14
P3	3.0	tau	134.805	26.72	13
P3	6.0	tau	119.589	34.76	12
P4	0.0	tau	130.336	32.23	15
P4	0.5	tau	147.686	34.05	14
P4	1.0	tau	172.787	41.64	15
P4	1.5	tau	160.081	40.17	15
P4	2.0	tau	150.638	30.13	14
P4	24.0	tau	129.142	32.13	15
P4	3.0	tau	135.021	31.60	14
P4	6.0	tau	124.516	25.77	14
P5	0.0	tau	119.477	26.11	15
P5	0.5	tau	191.724	58.37	15
P5	1.0	tau	192.202	45.19	15
P5	1.5	tau	161.129	33.28	14
P5	2.0	tau	163.966	32.56	14
P5	24.0	tau	113.212	20.27	15
P5	3.0	tau	136.903	38.72	15
P5	6.0	tau	128.459	35.93	15
P1	0.0	thre	113.530	19.85	15
P1	0.5	thre	123.382	23.00	15
P1	1.0	thre	121.530	21.91	15
P1	1.5	thre	175.467	43.35	15
P1	2.0	thre	142.070	27.12	14
P1	24.0	thre	125.839	24.52	15
P1	3.0	thre	104.407	22.46	15
P1	6.0	thre	98.705	15.76	15

protocol	time	analyte	mean	sd	n_obs
P2	0.0	thre	118.883	23.42	15
P2	0.5	thre	122.514	27.81	14
P2	1.0	thre	132.140	27.36	15
P2	1.5	thre	175.124	45.21	15
P2	2.0	thre	151.286	39.78	14
P2	24.0	thre	115.111	18.40	15
P2	3.0	thre	100.965	25.65	15
P2	6.0	thre	96.794	20.76	14
P3	0.0	thre	117.287	23.45	14
P3	0.5	thre	115.460	22.82	13
P3	1.0	thre	124.366	24.51	13
P3	1.5	thre	140.184	32.15	14
P3	2.0	thre	149.125	36.13	12
P3	24.0	thre	118.903	21.74	14
P3	3.0	thre	102.716	23.36	13
P3	6.0	thre	92.430	19.12	12
P4	0.0	thre	117.127	23.21	15
P4	0.5	thre	129.319	28.75	14
P4	1.0	thre	131.131	25.81	15
P4	1.5	thre	179.913	48.90	15
P4	2.0	thre	138.234	32.84	14
P4	24.0	thre	111.173	19.29	15
P4	3.0	thre	103.200	23.57	14
P4	6.0	thre	95.536	17.49	14
P5	0.0	thre	117.605	19.92	15
P5	0.5	thre	122.532	23.77	15
P5	1.0	thre	129.174	24.87	15
P5	1.5	thre	171.699	38.86	14
P5	2.0	thre	159.105	30.11	14
P5	24.0	thre	112.059	18.11	15
P5	3.0	thre	100.059	17.93	15
P5	6.0	thre	95.220	17.22	15
P1	0.0	trp	58.541	7.40	15
P1	0.5	trp	62.671	8.52	15

protocol	time	analyte	mean	sd	n_obs
P1	1.0	trp	60.946	6.91	15
P1	1.5	trp	57.989	6.89	15
P1	2.0	trp	53.432	5.10	14
P1	24.0	trp	59.871	8.83	15
P1	3.0	trp	51.865	5.07	15
P1	6.0	trp	52.782	5.85	15
P2	0.0	trp	64.367	9.72	15
P2	0.5	trp	70.021	9.38	14
P2	1.0	trp	77.427	10.50	15
P2	1.5	trp	71.329	8.94	15
P2	2.0	trp	66.874	9.08	14
P2	24.0	trp	55.389	6.26	15
P2	3.0	trp	52.788	7.85	15
P2	6.0	trp	53.530	5.39	14
P3	0.0	trp	59.710	7.31	14
P3	0.5	trp	63.926	8.47	13
P3	1.0	trp	68.867	7.36	13
P3	1.5	trp	67.081	7.63	14
P3	2.0	trp	65.838	7.28	12
P3	24.0	trp	55.545	7.32	14
P3	3.0	trp	53.574	4.96	13
P3	6.0	trp	51.325	7.15	12
P4	0.0	trp	62.611	7.83	15
P4	0.5	trp	69.582	7.57	14
P4	1.0	trp	72.244	7.34	15
P4	1.5	trp	64.756	8.70	15
P4	2.0	trp	61.096	7.71	14
P4	24.0	trp	61.311	7.23	15
P4	3.0	trp	55.459	7.72	14
P4	6.0	trp	56.159	7.44	14
P5	0.0	trp	61.812	6.73	15
P5	0.5	trp	67.944	9.26	15
P5	1.0	trp	70.791	5.91	15
P5	1.5	trp	67.312	6.67	14

protocol	time	analyte	mean	sd	n_obs
P5	2.0	trp	63.705	9.79	14
P5	24.0	trp	53.569	5.23	15
P5	3.0	trp	52.593	7.99	15
P5	6.0	trp	53.091	5.70	15
P1	0.0	tyr	59.207	7.50	15
P1	0.5	tyr	65.100	12.13	15
P1	1.0	tyr	64.245	8.54	15
P1	1.5	tyr	60.319	8.36	15
P1	2.0	tyr	54.645	6.01	14
P1	24.0	tyr	63.545	9.98	15
P1	3.0	tyr	49.233	7.45	15
P1	6.0	tyr	46.047	6.23	15
P2	0.0	tyr	60.167	9.17	15
P2	0.5	tyr	69.839	10.99	14
P2	1.0	tyr	81.021	13.40	15
P2	1.5	tyr	80.145	14.58	15
P2	2.0	tyr	75.100	15.12	14
P2	24.0	tyr	55.801	6.74	15
P2	3.0	tyr	60.360	12.50	15
P2	6.0	tyr	50.687	6.74	14
P3	0.0	tyr	57.621	8.28	14
P3	0.5	tyr	64.153	8.98	13
P3	1.0	tyr	71.300	10.51	13
P3	1.5	tyr	75.137	13.22	14
P3	2.0	tyr	73.032	13.95	12
P3	24.0	tyr	57.464	7.63	14
P3	3.0	tyr	59.232	8.57	13
P3	6.0	tyr	48.025	6.57	12
P4	0.0	tyr	59.997	8.85	15
P4	0.5	tyr	73.626	12.65	14
P4	1.0	tyr	78.548	11.36	15
P4	1.5	tyr	70.491	10.83	15
P4	2.0	tyr	64.346	10.32	14
P4	24.0	tyr	57.999	7.53	15

protocol	time	analyte	mean	sd	n_obs
P4	3.0	tyr	53.842	8.65	14
P4	6.0	tyr	47.897	5.76	14
P5	0.0	tyr	60.246	7.47	15
P5	0.5	tyr	69.611	8.09	15
P5	1.0	tyr	79.681	9.69	15
P5	1.5	tyr	79.097	9.07	14
P5	2.0	tyr	73.784	10.92	14
P5	24.0	tyr	54.869	7.09	15
P5	3.0	tyr	59.800	8.98	15
P5	6.0	tyr	48.645	5.84	15
P1	0.0	UREUM	4.520	1.02	15
P1	0.5	UREUM	4.533	1.04	15
P1	1.0	UREUM	4.540	1.03	15
P1	1.5	UREUM	4.807	1.08	14
P1	2.0	UREUM	5.233	1.01	15
P1	24.0	UREUM	5.113	1.16	15
P1	3.0	UREUM	5.313	0.97	15
P1	6.0	UREUM	4.860	0.91	15
P2	0.0	UREUM	4.100	0.79	15
P2	0.5	UREUM	4.379	0.69	14
P2	1.0	UREUM	4.480	0.78	15
P2	1.5	UREUM	5.260	0.84	15
P2	2.0	UREUM	5.779	0.64	14
P2	24.0	UREUM	5.233	0.85	15
P2	3.0	UREUM	5.920	0.81	15
P2	6.0	UREUM	5.264	0.73	14
P3	0.0	UREUM	4.921	1.01	14
P3	0.5	UREUM	5.261	0.92	13
P3	1.0	UREUM	5.300	1.04	14
P3	1.5	UREUM	5.529	1.06	14
P3	2.0	UREUM	6.233	0.95	12
P3	24.0	UREUM	5.414	1.04	14
P3	3.0	UREUM	6.879	0.94	14
P3	6.0	UREUM	6.409	0.90	11

protocol	time	analyte	mean	sd	n_obs
P4	0.0	UREUM	4.307	0.83	15
P4	0.5	UREUM	4.464	0.78	14
P4	1.0	UREUM	4.527	0.86	15
P4	1.5	UREUM	4.987	0.87	15
P4	2.0	UREUM	5.557	0.78	14
P4	24.0	UREUM	5.013	0.86	16
P4	3.0	UREUM	5.547	0.80	15
P4	6.0	UREUM	4.993	0.75	14
P5	0.0	UREUM	4.500	1.04	15
P5	0.5	UREUM	4.720	1.07	15
P5	1.0	UREUM	4.947	1.10	15
P5	1.5	UREUM	5.364	1.11	14
P5	2.0	UREUM	5.921	1.08	14
P5	24.0	UREUM	5.457	0.72	14
P5	3.0	UREUM	6.180	1.14	15
P5	6.0	UREUM	5.633	1.37	15
P1	0.0	val	210.186	19.00	15
P1	0.5	val	224.095	25.08	15
P1	1.0	val	219.326	20.17	15
P1	1.5	val	210.981	19.75	15
P1	2.0	val	196.701	23.32	14
P1	24.0	val	240.763	20.76	15
P1	3.0	val	188.041	19.26	15
P1	6.0	val	201.363	21.30	15
P2	0.0	val	215.245	17.65	15
P2	0.5	val	227.773	19.61	14
P2	1.0	val	237.535	22.43	15
P2	1.5	val	221.634	18.18	15
P2	2.0	val	205.915	20.08	14
P2	24.0	val	233.320	26.04	15
P2	3.0	val	186.058	19.30	15
P2	6.0	val	209.459	22.49	14
P3	0.0	val	216.467	28.97	14
P3	0.5	val	223.375	28.02	13

protocol	time	analyte	mean	sd	n_obs
P3	1.0	val	228.102	29.92	13
P3	1.5	val	227.349	29.12	14
P3	2.0	val	217.208	31.01	12
P3	24.0	val	224.069	23.59	14
P3	3.0	val	187.768	24.90	13
P3	6.0	val	200.863	20.71	12
P4	0.0	val	216.990	25.30	15
P4	0.5	val	239.795	24.66	14
P4	1.0	val	237.510	25.51	15
P4	1.5	val	217.047	23.91	15
P4	2.0	val	200.819	21.38	14
P4	24.0	val	231.987	29.18	15
P4	3.0	val	183.581	21.51	14
P4	6.0	val	201.750	20.84	14
P5	0.0	val	220.193	28.26	15
P5	0.5	val	229.465	29.69	15
P5	1.0	val	234.389	27.42	15
P5	1.5	val	224.003	32.07	14
P5	2.0	val	205.066	30.13	14
P5	24.0	val	238.481	28.79	15
P5	3.0	val	193.058	37.89	15
P5	6.0	val	219.103	48.74	15
P1	0.0	Zonulin	28.253	8.38	12
P1	1.0	Zonulin	31.286	8.80	12
P1	1.5	Zonulin	30.300	NA	1
P1	2.0	Zonulin	31.499	4.87	11
P1	24.0	Zonulin	31.820	6.25	12
P2	0.0	Zonulin	30.566	7.57	13
P2	1.0	Zonulin	34.672	4.82	13
P2	2.0	Zonulin	31.844	3.12	13
P2	24.0	Zonulin	33.849	4.44	13
P3	0.0	Zonulin	36.720	6.60	12
P3	0.5	Zonulin	33.210	NA	1
P3	1.0	Zonulin	37.092	6.01	12

protocol	time	analyte	mean	sd	n_obs
P3	1.5	Zonulin	42.890	0.35	2
P3	2.0	Zonulin	39.990	4.86	11
P3	24.0	Zonulin	36.287	9.61	12
P3	3.0	Zonulin	43.780	NA	1
P3	6.0	Zonulin	34.600	NA	1
P4	0.0	Zonulin	31.454	7.98	12
P4	1.0	Zonulin	33.087	7.37	12
P4	2.0	Zonulin	31.714	7.06	12
P4	24.0	Zonulin	30.561	5.86	12
